# Tertiary lymphoid structures correlate with the therapeutic efficacy and prognosis of resectable esophageal squamous cell carcinoma undergoing neoadjuvant chemoradiotherapy plus immunotherapy

**DOI:** 10.3389/fimmu.2025.1616247

**Published:** 2025-08-22

**Authors:** Ke Zhai, Ru Xie, Kun Ru, Miaoqing Zhao

**Affiliations:** ^1^ Shandong Cancer Hospital and Institute, Shandong First Medical University and Shandong Academy of Medical Sciences, Jinan, China; ^2^ Department of Pathology and Lab Medicine, Shandong Cancer Hospital and Institute, Shandong First Medical University and Shandong Academy of Medical Sciences, Jinan, China; ^3^ Department of Pathology, Shandong Cancer Hospital and Institute, Shandong First Medical University and Shandong Academy of Medical Sciences, Jinan, China

**Keywords:** tertiary lymphoid structures, esophageal squamous cell carcinoma, neoadjuvant therapy, immunotherapy, chemoradiotherapy, multiplex immunofluorescence, tumor biomarker, prognosis

## Abstract

**Background:**

Tertiary lymphoid structures (TLSs) are linked to prognosis in esophageal squamous cell carcinoma (ESCC), but whether the distribution, abundance, and maturity of TLSs affect therapeutic efficacy and prognosis in ESCC treated with neoadjuvant chemoradiotherapy plus immunotherapy (NRCI) remains unclear. We explored TLS characteristics and correlated them with patient survival.

**Methods:**

A total of 157 resectable ESCC patients treated with neoadjuvant therapy between September 2020 and May 2023 were divided into NRCI (n=49) and neoadjuvant chemoimmunotherapy (NCI, n=108) groups. Multiplex immunofluorescence (mIHC) was employed to compare the spatial distribution and cellular composition of TLSs in the NRCI (n=40) and NCI (n=40) groups. A TLSs scoring system assessed TLSs abundance and maturity across intratumoral regions (T regions), invasive margins (IM regions), and peritumoral regions (P regions). The differences in overall survival (OS) and disease-free survival (DFS) between the two groups were analyzed. Furthermore, whole-exome sequencing (WES) on 20 untreated ESCC samples examined the relationship between TLS infiltration and genetic mutations.

**Results:**

The OS and DFS in the NRCI group were significantly superior to the NCI group, with a higher rate of major pathological response (MPR). MPR patients exhibited significantly longer OS and DFS, suggesting that NRCI therapy substantially enhanced patient outcomes (all *P*<0.05). TLSs abundance exhibited varying immune effects in different tissue regions: intratumoral and invasive margin TLSs abundance was significantly associated with longer OS, while peritumoral TLSs abundance was linked to a shorter OS (all *P*<0.05). Highly mature TLSs (M-TLSs) were closely associated with a better OS (all *P*<0.05). In the NRCI group, M-TLSs showed higher proportions of CD20^+^Ki-67^+^ B cells, CD21^+^ dendritic cells (DCs), CD4^+^Ki-67^+^ helper T cells (Th), and CD8^+^Ki-67^+^ cytotoxic T cells compared to the NCI group (all *P*<0.05), indicating that NRCI therapy enhanced antitumor immune responses.

**Conclusion:**

NRCI therapy significantly enhanced the prognosis of resectable ESCCs compared to NCI therapy. The distribution and abundance of TLSs were clearly associated with OS in ESCCs and acted as independent prognostic indicators for OS in NRCI therapy. NRCI therapy extended OS and bolstered antitumor immune responses by facilitating the proliferation and activation of M-TLSs.

## 1 Introduction

The ESCC ranks as the seventh most common malignancy and the sixth leading cause of cancer-related mortality worldwide. As one of the most aggressive malignancies within the gastrointestinal tract, ESCC is characterized by its insidious nature and a 5-year survival rate of merely 22% (1). Despite radical surgery, most resectable ESCC cases are prone to recurrence and disease-related mortality, underscoring the pressing demand for innovative treatments. NRCI therapy combines radiotherapy, chemotherapy, and immunotherapy, representing one of the next frontiers in cancer therapy. It has been shown that NRCI therapy improved the pathological complete response (pCR) rate compared to NCI therapy (2), and the immune microenvironment has been shown to play a critical role in the progression of ESCC (3–5). The radiotherapy can modulate the immune microenvironment, thereby enhancing the efficacy of immunotherapy (6–8).

The successful application of immunotherapy in ESCC highlighted the necessity of better understanding the mechanisms of effective antitumor responses (9, 10). The infiltration of B and T cells within tumors has been widely investigated and has been found to improve prognosis in the majority of cancers (11–13). It has been shown that intratumoral B cells are positively associated with a favorable prognosis in ESCCs (14–16). Concurrently, several investigations have emphasized the importance of TLSs, defined as ectopic lymphoid aggregates formed in non-lymphoid tissues at inflamed sites associated with cancer, autoimmune conditions, and infections. TLSs were alternatively known as tertiary lymphoid organs (17, 18). TLSs provided an important microenvironment for humoral and cellular antitumor-specific immune responses by serving as local sites for antigen presentation and enabling the generation of effector and central memory T cells (19, 20). Growing evidence suggests that TLSs are strongly linked to improved prognosis in multiple cancers, including ESCC (13, 21–23). The induction of TLSs by neoadjuvant therapy has emerged as a critical factor in anti-tumor immunity and prognosis. In ESCC, the presence and maturation of TLSs have been associated with improved survival; however, prior study primarily examined TLSs in patients who received neoadjuvant chemotherapy, NCI, neoadjuvant chemoradiotherapy, or surgery alone, with M-TLSs serving as independent favorable prognostic factors across treatment groups (16). However, it is important to note that these analyses did not treat NRCI as a separate category, leaving unanswered whether combining radiotherapy, immunotherapy, and chemotherapy might further enhance TLSs-mediated anti-tumor immunity. Here, we address this gap by focusing on NRCI and its immunological and clinical effects on TLSs and ESCC.

Using two patient cohorts undergoing surgery after NRCI and NCI therapies, this study aimed to evaluate the spatial distribution, abundance, maturity, and cellular composition of TLSs, to investigate whether NRCI therapy impacts TLSs abundance and maturity and whether these TLSs characteristics influence the prognosis of ESCCs. The mIHC technology was employed to investigate the differences in the composition of M-TLSs located intratumorally, at invasive margins, and in peritumoral areas following NRCI and NCI therapies. Furthermore, this study developed a novel TLSs scoring system to assess the abundance and maturity of TLSs in intratumoral, invasive margin, and peritumoral regions, revealing that TLSs abundance exerted similar prognostic effects in intratumoral and invasive margin regions but opposite effects in peritumoral regions.

## 2 Materials and methods

### 2.1 Patients

Between September 2020 and May 2023, patients with resectable ESCC at Shandong Cancer Hospital (Shandong, China) were categorized into two cohorts based on preoperative treatments. One cohort consisted of 108 resectable ESCC patients who underwent surgery following NCI, and the other cohort included 49 resectable ESCC patients treated with NRCI. The inclusion criteria and specific treatment regimens are detailed in **Supplementary Figure 1**; **Table 1**. Clinicopathological features were collected. The MPR rate (MPR was defined as ≤10% residual viable tumor cells in the resected primary tumor specimen after neoadjuvant therapy), OS (OS was defined as the time from treatment to death or the last follow-up), and DFS (DFS was defined as the time from treatment without tumor, metastasis, or recurrence) were calculated. The median follow-up period was 37.5 months. Specimens from all patients were approved by the Ethics Committee of Cancer Hospital Affiliated to Shandong First Medical University (SDZLEC2024-041-02).

**Table 1:** Treatment regimens for different treatment groups.

Cohort	Treatment options	Patients number
NRCI	Tislelizumab 200mg + either Cisplatin 75mg/m^2^ or Carboplatin AUC 5 + Paclitaxel-albumin 135mg/m^2^ + RT: 1.8–2 Gy/F	40
	Camrelizumab 200mg + either Cisplatin 75mg/m^2^ or Carboplatin AUC 5 + Paclitaxel-albumin 135mg/m^2^ +RT: 1.8–2 Gy/F	9
NCI	Tislelizumab 200mg + either Cisplatin 75mg/m^2^ or Carboplatin AUC 5 + Paclitaxel-albumin 135mg/m^2^	86
	Camrelizumab 200mg + either Cisplatin 75mg/m^2^ or Carboplatin AUC 5 + Paclitaxel-albumin 135mg/m^2^	22

RT, radiotherapy; Gy, gray; F, fractions.

### 2.2 Histopathological and immunohistopathological analyses

**Figure 1:**
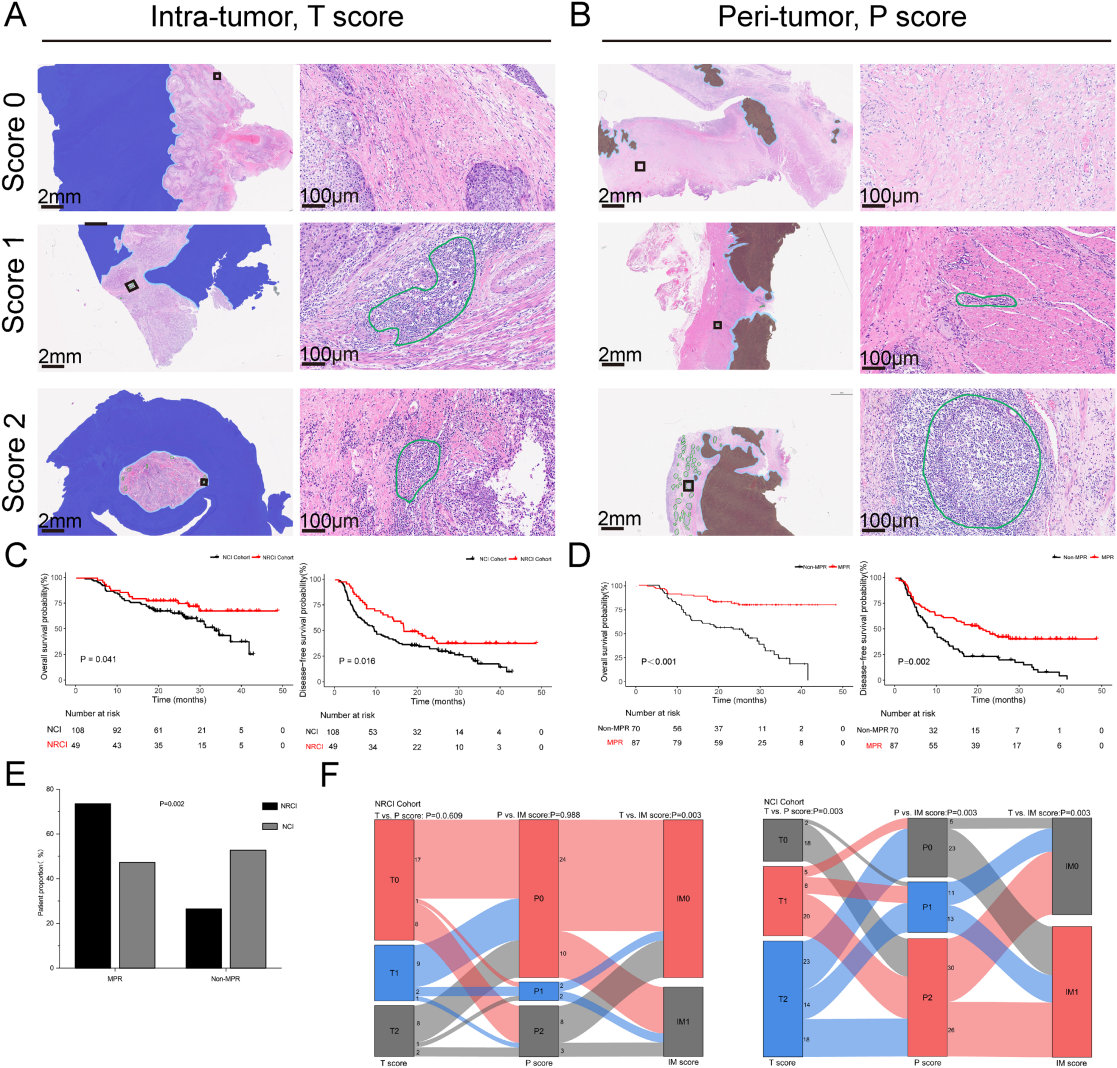
TLSs scoring system and the impact of NRCI therapy on prognosis and pathological response in ESCCs. **(A, B)** Representative whole slide images of the TLSs scoring system (T and P regions) using H&E staining. TLSs in intratumoral and peritumoral regions are marked in green. The intratumoral region (T region) is highlighted in light orange, while the peritumoral region (P region) is highlighted in deep blue. The invasive margin (IM region) is highlighted in light blue. **(C)** Kaplan-Meier curves showing OS and DFS in ESCCs from the NRCI and NCI cohorts. **(D)** Kaplan-Meier curves comparing OS and DFS between MPR and Non-MPR patients. **(E)** Comparison of the MPR rates among different treatment modalities (*P*= 0.002). **(F)** Correlation of T score, P score, and IM score between the NRCI and NCI cohorts. TLSs, tertiary lymphoid structures; ESCCs, esophageal squamous cell carcinoma patients; NRCI, neoadjuvant chemoradiotherapy plus immunotherapy; NCI, neoadjuvant chemoimmunotherapy.

**Figure 2:**
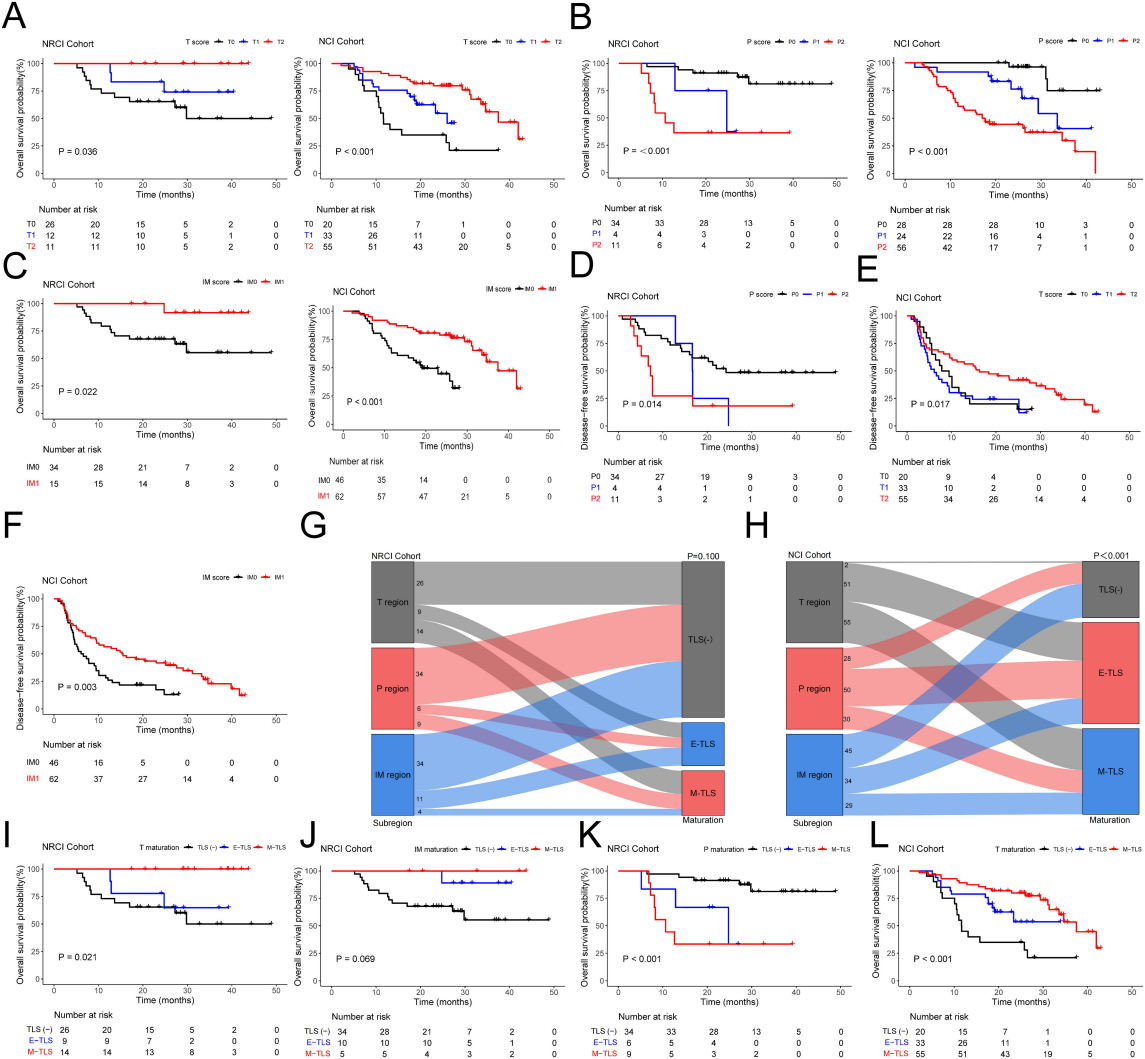
Kaplan-Meier survival analysis and the impact of TLS score and maturation on prognosis in ESCCs. **(A)** Kaplan-Meier curves showing OS in ESCCs from the NRCI and NCI cohorts stratified by T score. **(B)** Kaplan-Meier curves showing OS in ESCCs from the NRCI and NCI cohorts stratified by P score. **(C)** Kaplan-Meier curves showing OS in ESCCs from the NRCI and NCI cohorts stratified by IM score. **(D)** Kaplan-Meier curves showing DFS in ESCCs from the NRCI cohort stratified by P score. **(E)** Kaplan-Meier curves showing DFS in ESCCs from the NCI cohort stratified by T score. **(F)** Kaplan-Meier curves showing DFS in ESCCs from the NCI cohort stratified by IM score. **(G)** No significant association was observed between subregions and TLS maturation in the NRCI cohort (*P*= 0.100). **(H)** A significant association was observed between subregions and TLS maturation in the NCI cohort (*P*< 0.001). **(I)** Kaplan-Meier curves showing OS in ESCCs from the NRCI cohort stratified by T maturation. **(J)** Kaplan-Meier curves showing OS in ESCCs from the NRCI cohort stratified by IM maturation. **(K)** Kaplan-Meier curves showing OS in ESCCs from the NRCI cohort stratified by P maturation. **(L)** Kaplan-Meier curves showing OS in ESCCs from the NCI cohort stratified by T maturation.

For each case in the NRCI and NCI groups, slides were blindly evaluated by two independent pathologists. For spatial characterization and quantification of TLSs, the morphological assessment of TLSs was performed on H&E-stained slides, which were scanned into whole slide images (WSIs) using the NanoZoomer S210 Digital Pathology Scanning System (Hamamatsu, Hamamatsu City, Shizuoka, Japan). Briefly, TLSs were categorized according to maturation stages as TLS negative (TLS(-), regarded as the initial stage or absence of TLS development), early TLS (E-TLS, an aggregate of B and T cells in the absence of follicular dendritic cell infiltration and without germinal centers), and mature TLS (M-TLS, a follicle-like TLS composed of B cells, T cells, and follicular dendritic cells, with or without germinal centers). To assess TLSs spatial distribution, WSIs were segmented into three subregions: intra-tumor (T), peri-tumor (P), and invasive margin (IM, a 0.1 mm wide zone on both sides of the boundary between intra-tumor and peri-tumor regions) (**Supplementary Methods**; **Figure 2A**). A TLSs scoring system was established based on TLSs density in different subregions. In the T region (T score), TLSs density was classified into three groups: (1) score 0: no TLS in the T region; (2) score 1: 1 or 2 TLSs in the T region; (3) score 2: at least 3 TLSs, either converging or non-converging throughout the tumor region. TLSs density in the P region (P score) followed the same scoring criteria. TLSs density in the IM region (IM score) was categorized into two groups, as shown in the **Supplementary Methods**; **Figure 2B**. Representative WSIs of the TLSs scoring system are shown in **Figures 1A, B**. In order to reduce inter-observer bias, TLSs scores were independently evaluated by two senior pathologists who were blinded to the clinical data. To ensure the accuracy of the TLSs scoring system, TLSs density (number of TLSs per scoring subregion area/area of scoring subregion) was calculated in the NRCI cohort, and the results indicated that the TLSs scoring system essentially represented TLSs density (**Supplementary Figure 2C**). Subsequently, TLSs scoring was performed in the NRCI and NCI cohorts, with the higher score adopted when discrepancies arose between the two independent pathologists (**Supplementary Figures 2D–L**). Additional information about the TLSs scoring system was provided in the **Supplementary Methods**.

### 2.3 Whole-exome sequencing

WES was conducted to explore whether highly prevalent mutations can predict TLSs infiltration and maturation status after NRCI therapy. The genomic DNA was extracted from pre-neoadjuvant biopsy tumor samples of 20 ESCCs using the AllPrep DNA/RNA FFPE Kit (Qiagen, Hilden, Germany) per the manufacturer’s protocol. The KAPA DNA Library Preparation Kit (Kapa Biosystems, Wilmington, MA, USA) was used to prepare sequencing libraries for gDNA. The libraries were then hybridized with the xGen^®^ Exome Research Panel (Integrated DNA Technologies, Coralville, IA, USA). The whole-exome sequencing was performed using the GeneSeq-2000 platform (Geneplus-Suzhou, Suzhou, China). Adapter sequences and low-quality reads were removed from the raw sequencing data. High-quality reads were selected based on a Phred score ≥30, a mapping quality score ≥30, and no bias in paired-end reads. Clean reads were aligned to the human reference genome (hg19) using BWA (version 0.7.15-r1140). Picard (version 1.98) was used to mark PCR duplicates. GATK (version v3.6-0-g89b7209) was employed for the realignment and recalibration of redundant reads. DNA from adjacent normal tissue served as the germline control. Twenty patients were categorized by the abundance and maturity of TLSs. Fisher’s exact test was applied to assess the significance of mutation incidence differences between groups, indicating potential correlations between specific mutations and TLSs scores.

### 2.4 mIHC and data analysis

To investigate the cellular composition of spatially distinct TLSs and explore the potential mechanisms by which NRCI therapy improves patient prognosis via TLSs, the mIHC was conducted on 40 cases in the NRCI cohort and 40 in the NCI cohort, with each cohort comprising 20 MPR and 20 Non-MPR patients. As described earlier, mIHC was performed using the Pheno Multicolor Immunofluorescence Detection Kit (PhenoVision Bio Co., Ltd) according to the manufacturer’s instructions (**Supplementary Table 1**). The tissue sections underwent dewaxing and hydration before being transferred to a PH 6.0 retrieval buffer for antigen retrieval. The sections were washed and incubated with H2O2 and blocking agents for 10 minutes each. The primary antibodies (Ki67, PanCK, CD20, CD21, CD8, and CD4) were added to each section and incubated at room temperature for 30 minutes. The secondary antibodies (PVB anti-Rb/Mm-HRP) from the kit were used to detect the primary antibodies, followed by labeling with PVB amide signal amplification fluorophores (PVB480, PVB520, PVB570, PVB620, or PVB690). The slides were stained with DAPI (4′,6-diamidino-2-phenylindole) and sealed with anti-fluorescence quenching mounting medium.

The slides were then scanned at 20x magnification using the PhenoImage HT system (Akoya Biosciences). Visiopharm software 2023.09 (Hoersholm, Denmark) was employed for image analysis. First, invasive margins were outlined in the WSIs as 200 μm regions centered on the interface of the tumor and adjacent tissues. The tumor areas were identified via PanCK positivity and morphological features, with lymphocyte counts and proportions analyzed across regions. Secondly, TLSs were identified based on B-cell clustering and tissue morphology, with CD21 used to differentiate maturity: CD21-negative TLS was classified as E-TLS, and CD21-positive TLS as M-TLS. The number and proportion of lymphocytes within TLSs were then quantified.

### 2.5 Statistical analysis

The statistical analyses were conducted using SPSS V.27.0 (IBM) and R 4.4.2. The categorical variables were evaluated using χ2 test, Fisher’s exact test, or Kruskal-Wallis test. The continuous variables were analyzed using Student’s t-test or Mann-Whitney U test, and the results were reported as mean with SD or medians and IQR. The Pearson correlation coefficient was used to evaluate the correlation between two continuous variables. The survival curve analysis was performed using the Kaplan-Meier method and the log-rank test. Univariate and multivariate cox regression analyses, along with regularization techniques, were used to evaluate independent factors associated with OS and DFS. The results of cox regression analysis were presented as hazard ratios (HR) with 95% confidence intervals (CI). P values were adjusted for multiple testing using the false discovery rate (FDR) correction according to the Benjamini-Hochberg (BH) method. The cut-off values for converting continuous variables into categorical variables were determined via ROC curve analysis or median values. The two-sided p-values <0.05 were regarded as statistically significant.

## 3 Results

### 3.1 Clinicopathological characteristics of ESCCs

FFPE tissues from 157 resected ESCCs were retrospectively collected, including those treated with NCI (n=108) and NRCI (n=41). The clinicopathological characteristics of the patients were summarized in **Table 2**. The median DFS for the NRCI and NCI groups were 16.73 months (range: 0.90–48.87) and 14.20 months (range: 0.93–43.00), respectively. The median OS in the NRCI group was 27.40 months (range: 5.17–48.87), compared to 23.15 months (range: 2.13–43.00) in the NCI group. In the NRCI cohort, 73.5% (n=36) of patients achieved MPR, and 16 of them achieved pCR. Among them, 96 patients (88.9%) were male, with a median age of 59 years (IQR 48–71). In the NCI group, 47.2% (n=51) achieved MPR, including 24 patients with pCR. Among them, 41 patients (83.7%) were male, with a median age of 63 years (IQR 45–74). There were no statistically significant differences between the two cohorts in terms of gender, AJCC/UICC 8th cTNM stage, AJCC/UICC 8th ypTNM stage, lymph node metastasis, differentiation, vascular invasion, or tumor necrosis.

**Table 2:** Demographics and clinicopathologic characteristics of patients with ESCC.

Characteristics	NCI Cohort	NRCI Cohort	P value (95%CI)
	(n=108)	(n=49)	
Gender			
Male(%)	96(88.9)	41(83.7)	0.364^*^
Female(%)	12(11.1)	8(16.3)	
Age, years			
Mean(SD)	63.0(6.7)	59.4(5.6)	0.001
Range	(45.0-74.0)	(48.0-71.0)	
MPR			
Yes (%)	51(47.2)	36(73.5)	0.002^*^
No (%)	57(52.8)	13(26.5)	
AJCC/UICC 8th cTNM stage			
II(%)	33(30.6)	12(24.5)	0.600
III(%)	59(54.6)	30(61.2)	
VI(%)	16(14.8)	7(14.3)	
AJCC/UICC 8th ypTNM stage			
I(%)	53(49.0)	29(59.2)	0.466
II(%)	14(13.0)	3(6.1)	
III(%)	33(30.6)	12(24.5)	
VI(%)	8(7.4)	5(10.2)	
TRG			
0(%)	32(29.6)	20(40.8)	0.021
1(%)	19(17.6)	13(26.5)	
2(%)	34(31.5)	12(24.5)	
3(%)	23(21.3)	4(8.2)	
Mild-to-moderate myelosuppression			
Yes (%)	12(11.1)	18(36.7)	<0.001^*^
No (%)	96(88.9)	31(63.3)	
Neoadjuvant therapy no of cycles			
1(%)	2(1.9)	15(30.6)	<0.001
≥2(%)	106(98.1)	34(69.4)	
Lymph node metastasis			
Positive (%)	41(38.0)	21(57.1)	0.561^*^
Negative (%)	67(62.0)	28(42.9)	
Differentiation			
Well (%)	8(7.4)	0(0)	0.940
Moderate (%)	73(67.6)	40(81.6)	
Poor (%)	27(25)	9(18.4)	
Vascular invasion			
Yes (%)	20(18.5)	5(10.2)	0.187^*^
No (%)	88(81.5)	44(89.8)	
Nerve infiltration			
Positive (%)	16(14.8)	2(4.1)	0.039^†^
Negative(%)	92(85.2)	47(95.9)	
Tumor necrosis			
Positive (%)	5(4.6)	17(34.7)	<0.001^*^
Negative(%)	103(95.4)	32(65.3)	
Tumor necrosis			
Mean(SD)	0.1(0.8)	3.8(8.8)	<0.001
Range	0.0(6.9)	0.0(52.5)	

CI, confidence interval; NRCI, chemoradiotherapy plus immunotherapy; NCI, neoadjuvant chemoimmunotherapy.

*Chi-square test, †Fisher’s exact test, Mann-Whitney U test was used for other tests.

### 3.2 The prognosis was significantly better for patients treated with NRCI

**Figure 1** showed the prognoses of patients in the two cohorts, with the NRCI group demonstrating significantly longer DFS and OS compared to the NCI group (**Figure 1C**, all *P*<0.05). A combined analysis of 157 patients from both cohorts revealed that DFS and OS were significantly prolonged in the MPR group compared to the Non-MPR group (**Figure 1D**, all *P*<0.001). Additionally, the MPR rate was significantly higher in the NRCI group than in the NCI group (**Figure 1E**, *P*=0.002). These findings indicated that patients receiving NRCI therapy had better prognoses than those receiving NCI therapy. It suggested that radiotherapy might enhance the efficacy of immunotherapy. The improved MPR rate may result from the synergistic effect of radiotherapy’s direct cytotoxicity against tumor cells and its immune-activating function.

### 3.3 TLSs in different subregions are associated with distinct prognoses in ESCCs

**Figure 1F** illustrated the T, P, and IM scores in the two cohorts. In the NRCI group, no significant correlation was observed between T and P scores or between P and IM scores, indicating that TLS density in tumor and peritumoral areas, as well as invasive margins and peritumoral areas, were independent of each other (all *P*>0.05). A significant correlation was found between T and IM scores, indicating that TLS densities in the tumor region and invasive margins were interrelated (*P*=0.003, adjusted by BH). In contrast, the significant correlations among T, P, and IM scores in the NCI group further confirmed the intrinsic associations between TLS densities across subregions (all *P*<0.01).

We evaluated TLSs scores in three different subregions within the NRCI and NCI cohorts, and the results demonstrated distinctly different prognoses. The T scores showed a positive correlation with OS (**Figure 2A**, all *P*<0.05); P scores were negatively correlated with OS (**Figure 2B**, all *P*<0.001); IM scores were positively correlated with OS (**Figure 2C**, all *P*<0.05). Additionally, the survival analysis was conducted to assess the correlation between TLSs scores in various subregions and DFS in both cohorts. The results showed that in the NRCI group, P scores were negatively correlated with DFS (**Figure 2D**, *P*=0.014). In contrast, T scores in the NCI group were positively correlated with DFS (**Figure 2E**, *P*=0.017), and IM scores were also positively correlated with DFS (**Figure 2F**, P=0.003). Other subregions were not associated with DFS in either cohort (**Supplementary Figure 3A**, all *P*>0.05). These findings suggested that TLSs scores in different subregions had potential predictive value for OS and DFS in ESCCs. Furthermore, TLS maturity stages were evaluated in the NRCI and NCI cohorts, revealing no significant associations between subregions and TLS maturity stages in the NRCI cohort (**Figure 2G**, *P*=0.100); however, significant associations were found between subregions and TLS maturity stages in the NCI cohort (**Figure 2H**, *P*< 0.001). These findings indicated that NRCI enhanced the independence of TLS maturity stages across subregions.

**Figure 3:**
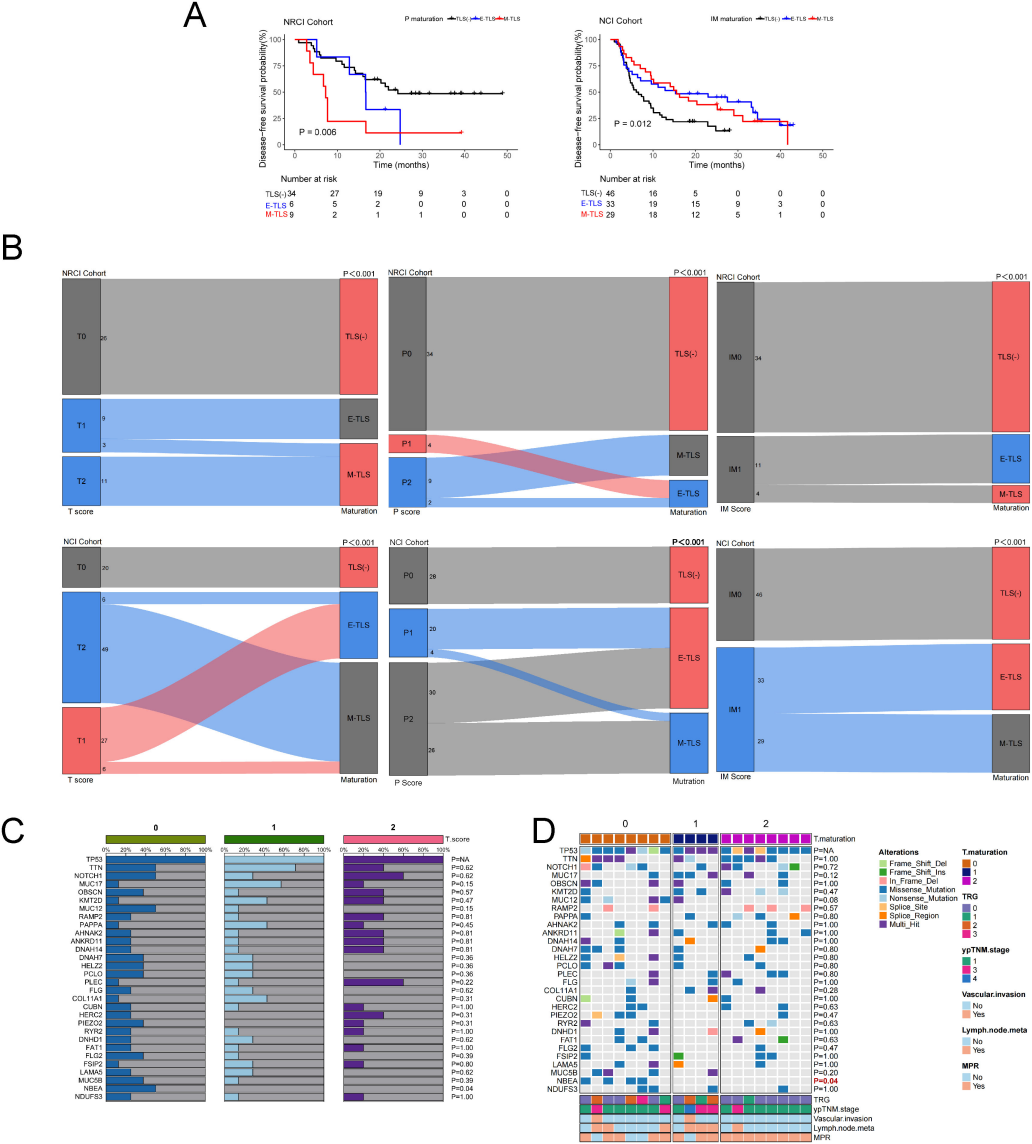
Correlation of TLS maturation with ESCC prognosis, TLS score, TLS maturation, and T region TLS with gene mutations. **(A)** Kaplan-Meier curves showing DFS in ESCCs from the NRCI cohort stratified by P maturation and DFS in the NCI cohort stratified by IM maturation. **(B)** TLS maturation stage is significantly positively correlated with T, P, and IM scores (all *P*< 0.001). **(C)** A potential association was observed between NBEA mutation (n=4) and T score (*P*= 0.037). **(D)** A potential association was observed between NBEA mutation (n=4) and T maturation (*P*= 0.042).

Furthermore, we found that in the T region of the NRCI cohort, ESCCs with M-TLSs had prolonged OS compared to TLS-negative and E-TLSs ESCCs (**Figure 2I**, *P*=0.021). The maturation stage of TLSs in the IM region showed results consistent with the above findings (**Figure 2J**, *P*=0.069, borderline significance). In contrast, ESCCs with M-TLSs had shorter OS than TLS-negative and E-TLSs ESCCs in the P region (**Figure 2K**, *P*<0.001). This indicated that the higher maturity of T and IM regions was associated with a better prognosis, whereas the higher maturity in the P region was associated with a poorer prognosis in ESCCs. In the NCI cohort, the TLSs maturation stage in the T region showed results consistent with the NRCI cohort (**Figure 2L**, *P*< 0.001). ESCCs with E-TLSs had a shorter OS compared to TLS-negative and M-TLSs ESCCs in the P region, whereas ESCCs with E-TLSs had a prolonged OS in the IM region (**Supplementary Figure 3B**, all *P*< 0.001). At the same time, we performed survival analysis for DFS based on the TLS maturation stages in different subregions of the two cohorts. It was demonstrated that the P maturation in the NRCI cohort and the IM maturation in the NCI cohort were consistent in predicting DFS as well as OS (**Figure 3A**, all *P*< 0.05). However, in both cohorts, no significant association was found between the TLS maturation stage in other subregions and DFS (**Supplementary Figure 3C**, all *P*>0.05). At the same time, it was found that the TLS maturation grade was positively correlated with T, P, and IM scores (**Figure 3B**, all *P*<0.001). In addition, we used WES gene sequencing to explore the predictive ability of high-frequency mutations on TLSs infiltration and maturation after NRCI and found: 1. In the NRCI cohort (n=20), *TP53* mutations were present in all individuals, making it the most frequently mutated gene. 2. Identified were recurrent genetic alterations (*TTN, NOTCH1, MUC17, OBSCN, KMT2D, MUC12, AHNAK2, PCLO, HERC2, RYR2, MUC5B*), tumor progression-related genes (*RAMP2, PAPPA, HELZ2, PIEZO2, DNHD1, NBEA*), repair and stability regulation genes (*ANKRD11, DNAH14, DNAH7, FSIP2, FLG, FLG2, PLEC*), microenvironment regulation and matrix remodeling genes (*CUBN, LAMA5, COL11A1*), and metabolism and mitochondrial function-associated genes (*NDUFS3, DNHD1, FSIP2*). All 20 samples included tumor tissues before neoadjuvant therapy and after surgical resection, along with corresponding H&E sections of the adjacent esophageal tissue. These 20 samples were included in the correlation analysis. 3. Except for the potential associations between *NBEA* mutation (n=4) and T scores (**Figure 3C**, *P*=0.037) and between *NBEA* mutation (n=4) and T maturity (**Figure 3D**, *P*=0.042), no other correlations were observed between TLSs infiltration and maturation and these mutations in ESCCs following NRCI (**Supplementary Figures 4A–D**, all *P*>0.05). 4. While *NBEA* mutation seemed to correlate with T scores and T maturity, the role of genetic mutations in tumor-associated immunity needs further investigation, considering the limited number of cases with *NBEA* mutation (n=4).

### 3.4 Cox regression models for TLSs in different subregions and their predictive roles for OS and DFS

We further employed regularization techniques combined with cox regression to assess how TLSs abundance and maturity in different subregions predicted OS and DFS in the two patient cohorts. The entire cohort had a median follow-up time of 37.5 months. In the NRCI group, 28.6% (n=14) died, while in the NCI group, 43.5% (n=47) succumbed. In the NRCI group, 61.2% (n=30) experienced recurrence or progression, compared to 76.9% (n=83) in the NCI group. Across both cohorts, the high-maturity and high-abundance TLSs groups showed superior OS compared to the low-maturity and low-abundance groups (**Figure 2**). Due to severe multicollinearity issues with T score (VIF=14.77), T maturity (VIF=14.34), P score (VIF=45.04), and P maturity (VIF=41.90) in the NRCI cohort, we used elastic net regression to analyze variables related to OS. This approach excluded P maturity from the multivariable analysis. Similarly, when analyzing variables related to DFS, P score (VIF=21.72) and P maturity (VIF=20.92) showed severe multicollinearity, leading to exclude P maturity from the multivariable analysis in the NRCI cohort to enhance model consistency. The multivariate analysis identified the abundance of TLSs in the peritumoral region as an independent predictor of OS in ESCCs (NRCI cohort: *P*=0.003, adjusted by BH; NCI cohort: *P*=0.015, adjusted by BH) (**Table 3**). Furthermore, we used regularization techniques and cox regression to analyze the association of TLSs abundance and maturity in different subregions with DFS. Consistent with prior correlation analyses and Kaplan-Meier results, we did not identify independent predictors for DFS, possibly due to the small sample size, highlighting the need for larger cohorts to validate the predictive roles of TLSs abundance and maturity for DFS (**Supplementary Table 2**).

**Table 3:** Univariate and multivariate analysis of OS for the two different groups.

Variables	NCI cohort(n=108)				NRCI cohort(n=49)			
	Univariate analysis		Multivariate analysis		Univariate analysis		Multivariate analysis	
	*HR* (95%*CI*)	*P* value	*HR* (95%CI)	*P* value	*HR* (95%*CI*)	*P* value	*HR* (95%*CI*)	*P* value
Gender (Male or female)	0.32(0.08-1.34)	0.160			0.87(0.41-1.84)	0.750		
Age, years <64 vs ≥64	0.68(0.38-1.21)	0.229			0.52(0.12-2.31)	0.459		
MPR (No vs. Yes)	0.25(0.13-0.51)	0.002	0.63(0.16-2.42)	0.714	0.30(0.10-0.85)	0.047	1.08(2.58-3.40)	0.999
AJCC/UICC 8th cTNM stage		0.543				0.606		
II	Reference				Reference			
III	0.77(0.40-1.46)	0.479			2.04(0.44-9.43)	0.444		
VI	1.22(0.52-2.84)	0.683			2.61(0.44-15.64)	0.369		
AJCC/UICC 8th ypTNM stage		0.002		0.354				0.999
I	Reference				Reference			
II	1.92(0.76-4.81)	0.209	0.19(0.04-0.95)	0.213	0.00(0.00-)	0.985	0.75(1.79-2.62)	0.045
III	3.03(1.50-6.11)	0.004	0.21(0.04-1.08)	0.213	4.67(1.31-16.61)	0.041	1.28(3.29-3.95)	0.003
VI	8.82(3.44-22.61)	0.002	0.36-0.05-2.66)	0.634	9.05(2.23-36.69)	0.008	1.32(3.23-4.47)	0.003
Mild-to-moderate myelosuppression (No vs. Yes)	0.71(0.26-1.99)	0.562			1.41(0.48-4.09)	0.591		
Neoadjuvant therapy no of cycles (1 vs. ≥2)	0.80(0.11-5.84)	0.846			1.23(0.38-3.92)	0.751		
Lymph node metastasis (No vs. Yes)	4.13(2.27-7.52)	0.002	3.48(0.93-13.08)	0.213	6.70(1.86-24.18)	0.013	1.35(3.55-4.25)	0.410
Differentiation		0.200				0.369		
Well	Reference				–			
Moderate	2.16(0.51-9.08)	0.347			Reference			
Poor	3.44(0.78-15.25)	0.143			0.33(0.04-2.55)	0.369		
Vascular invasion (No vs. Yes)	2.64(1.38-5.03)	0.006	1.27(0.46-3.48)	0.757	17.94(4.66-69.10)	0.006	1.87(4.98-8.89)	0.003
Nerve infiltration (No vs. Yes)	2.91(1.41-6.00)	0.007	2.02(0.67-6.07)	0.522	4.34(0.96-19.65)	0.093		
Tumor necrosis (<1.43 vs. ≥1.43)	3.59(1.25-10.33)	0.029	0.67(0.16-2.75)	0.757	2.02(0.70-5.85)	0.269		
TRG		0.002		0.714		0.114		0.999
0	Reference				Reference			
1	0.96(0.27-3.41)	0.948	1.32(0.30-5.82)	0.769	1.78(0.36-8.83)	0.552	0.98(2.44-2.94)	0.008
2	3.35(1.34-8.38)	0.017	2.47(0.50-12.09)	0.570	3.56(0.84-14.99)	0.121	1.06(2.14-4.40)	0.108
3	5.15(2.02-13.08)	0.002	4.31(0.65-28.52)	0.355	7.68(1.51-39.21)	0.036	1.31(2.89-4.99)	0.036
TLSs maturation								
T region		0.002		0.714		0.041		0.999
TLSs(-)	Reference				Reference			
E-TLSs	0.47(0.22-0.99)	0.070	0.64(0.10-3.96)	0.757	1.31(1.08-1.59)	0.020	0.95(2.10-3.42)	0.945
M-TLSs	0.24(0.12-0.48)	0.002	1.73(0.36-8.36)	0.714	2.61(2.15-3.18)	0.006	0.76(2.02-2.25)	0.003
P region		0.002		0.015		0.011		
TLSs(-)	Reference				Reference	0.008		
E-TLSs	10.45(3.13-34.86)	0.002	18.02(4.01-81.06)	0.015	5.50(1.26-23.97)	0.047		
M-TLSs	6.30(1.91-21.99)	0.007	4.24(0.98-18.36)	0.213	8.20(2.44-27.53)	0.006		
IM region		0.002		0.714		0.015		0.401
TLSs(-)	Reference				Reference			
E-TLSs	0.17(0.07-0.41)	0.002	0.39(0.08-1.81)	0.522	0.36(0.29-0.43)	0.006	0.80(2.11-2.34)	0.003
M-TLSs	0.39(0.19-0.82)	0.022	0.70(0.15-3.35)	0.757	2.97(2.44-3.61)	0.006	0.71(1.87-2.24)	0.003
TLSs abundance								
T score		0.002		0.757		0.047		0.999
0	Reference				Reference			
1	0.52(0.25-1.08)	0.113	1.37(0.31-6.06)	0.757	1.22(1.01-1.49)	0.075	0.88(2.20-2.68)	0.045
2	0.22(0.11-0.45)	0.002	1.00(0.15-6.27)	0.714	0.55(0.45-0.67)	0.006	0.79(2.02-2.43)	0.003
P score		0.002		0.015		0.008		0.003
0	Reference				Reference			
1	4.24(1.12-16.11)	0.052	0.20(0.07-0.56)	0.015	4.61(0.87-24.58)	0.114	1.07(2.66-3.25)	0.219
2	10.81(3.31-35.32)	0.002	22.07(4.92-98.86)	0.213	8.31(2.57-26.87)	0.006	1.48(2.90-7.94)	0.042
IM score		0.002		0.880		0.069		0.999
0	Reference				Reference			
1	0.28(0.14-0.54)	0.002	1.08(0.23-5.16)	0.880	0.12(0.02-0.89)	0.069	0.77(1.87-2.58)	0.035

P-values between 0.05 and 0.10 were considered borderline significant.

P values were FDR-adjusted using the BH method.

OS, overall survival; HR, hazard ratio; CI, confidence interval; NRCI, chemoradiotherapy plus immunotherapy; NCI, neoadjuvant chemoimmunotherapy; M-TLSs, highly mature TLSs; FDR, the false discovery rate; BH, Benjamini-Hochberg method.

### 3.5 Immune cell composition levels of TLSs in different subregions

**Figure 4:**
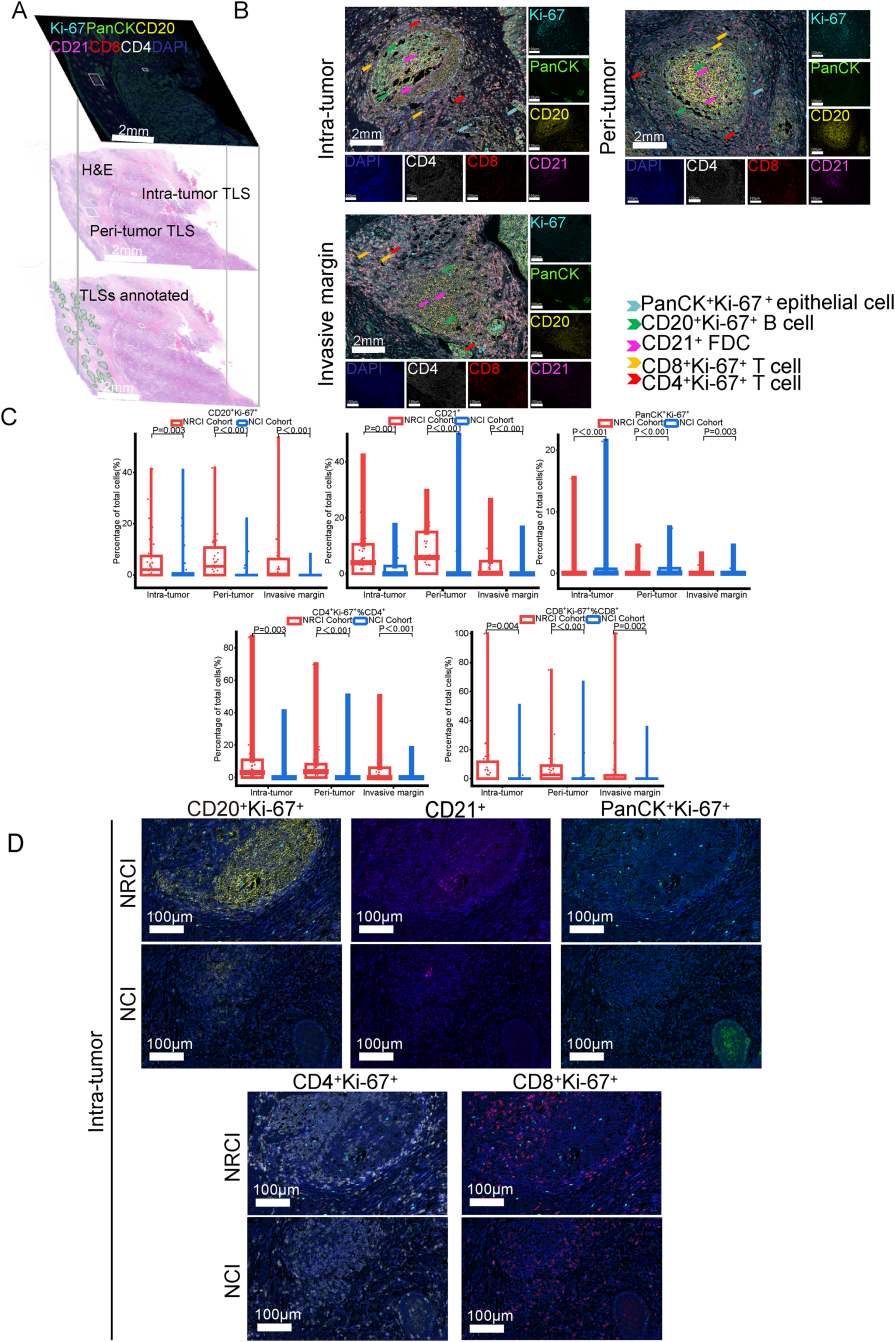
Differences in cellular composition of M-TLSs in the NRCI and NCI cohorts with identical subregions (intratumoral, invasive margin, and peritumoral areas). **(A)** Spatially matched images of TLSs in ESCCs, including TLS annotations with H&E staining and mIHC staining (CD20, CD21, CD4, CD8, Ki-67, PanCK). **(B)** Representative mIHC images showing positive expression of CD20, CD21, CD4, CD8, Ki-67, and PanCK in TLSs located in intratumoral, peritumoral, and invasive margin regions. Scale bar, 100 µm. **(C)** Quantification of M-TLSs in intratumoral, peritumoral, and invasive margin regions with identical subregions in the NRCI and NCI cohorts. The percentages of CD20^+^Ki-67^+^ B cells, CD21^+^ DCs, PanCK^+^Ki-67^+^ epithelial cells, CD8^+^Ki-67^+^ cytotoxic T lymphocytes, and CD4^+^Ki-67^+^ Th cells were analyzed for each subregion. **(D)** Representative mIHC images showing staining of CD20, CD21, PanCK, Ki-67, CD8, and CD4 in intratumoral M-TLSs from the NRCI and NCI cohorts. Scale bar, 50 µm. mIHC, multiplex immunohistochemistry; M-TLSs, mature tertiary lymphoid structures; DCs, dendritic cells.

**Figure 5:**
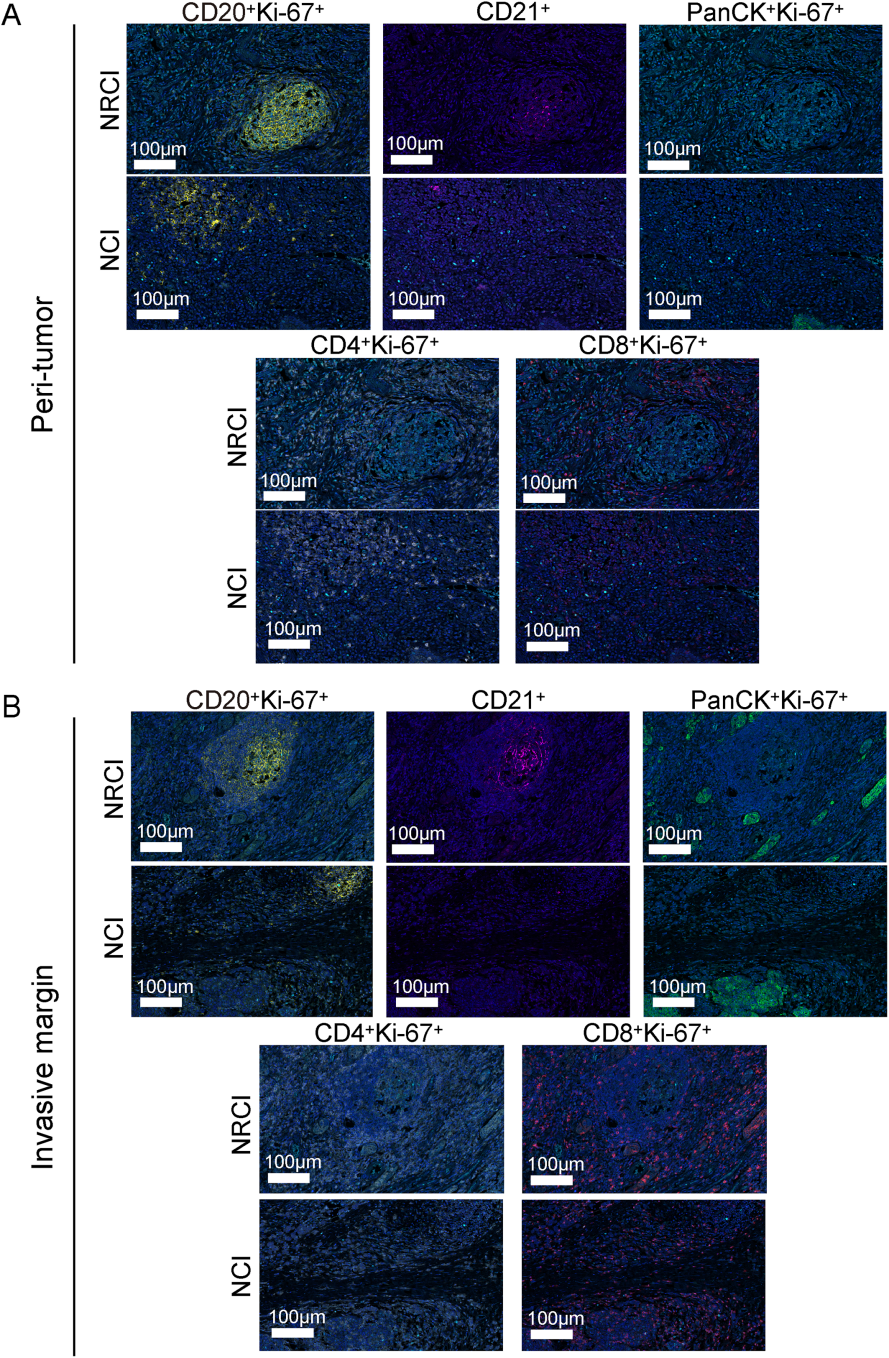
Differences in immune cell composition of M-TLSs in the NRCI and NCI cohorts under identical TLS scores (P score and IM score). **(A)** Representative mIHC images showing staining of CD20, CD21, PanCK, Ki-67, CD8, and CD4 in peritumoral M-TLSs from the NRCI and NCI cohorts. Scale bar, 100 µm. **(B)** Representative mIHC images showing staining of CD20, CD21, PanCK, Ki-67, CD8, and CD4 in invasive margin M-TLSs from the NRCI and NCI cohorts. Scale bar, 100 µm. mIHC, multiplex immunohistochemistry; M-TLSs, mature tertiary lymphoid structures; DCs, dendritic cells.

To explore the potential mechanisms by which NRCI improves patient prognosis via TLSs, we included 40 ESCC samples from the NRCI cohort and 40 ESCC samples from the NCI cohort, staining the slides with H&E and mIHC (**Figure 4A**). We then analyzed the impact of different treatment modalities on the cellular composition of M-TLSs within the same subregions (tumor interior, invasive margin, and peritumoral area). Among six types of immune cells, including Th cells (CD4^+^), cytotoxic T cells (CD8^+^), B cells (CD20^+^), DCs (CD21^+^), proliferating cells (Ki67^+^), and epithelial cells (PanCK^+^) (**Figure 4B**), the distributions of proliferating B cells (CD20^+^Ki-67^+^), DCs (CD21^+^), proliferating Th cells (CD4^+^Ki-67^+^), proliferating cytotoxic T cells (CD8^+^Ki-67^+^), and proliferating epithelial cells (PanCK^+^Ki67^+^) differed significantly between the NRCI and NCI groups. Specifically, the proportions of CD20^+^Ki-67^+^ B cells and CD21^+^ DCs were significantly higher, while those of PanCK^+^Ki-67^+^ epithelial cells were significantly lower, within the same subregions, in the NRCI group compared to the NCI group (**Figures 4C, D**, **5A, B**, percentage: all *P*<0.01). Additionally, the proportions of CD8^+^Ki-67^+^ cytotoxic T cells within total CD8^+^ cytotoxic T cells in each region and CD4^+^Ki-67^+^ Th cells within total CD4^+^ Th cells in each region were increased across all regions (**Figures 4C, D**, **5A, B**, percentage: all *P*<0.01). It was observed that the activation and infiltration of immune cells in M-TLSs were higher in the NRCI group than in the NCI group across all subregions, indicating that NRCI treatment effectively enhanced antitumor immune responses in ESCCs. Meanwhile, epithelial cell proliferation in M-TLSs was markedly lower in the NRCI group compared to the NCI group, implying that the NRCI could enhance treatment efficacy by reducing tumor aggressiveness.

**Figure 6:**
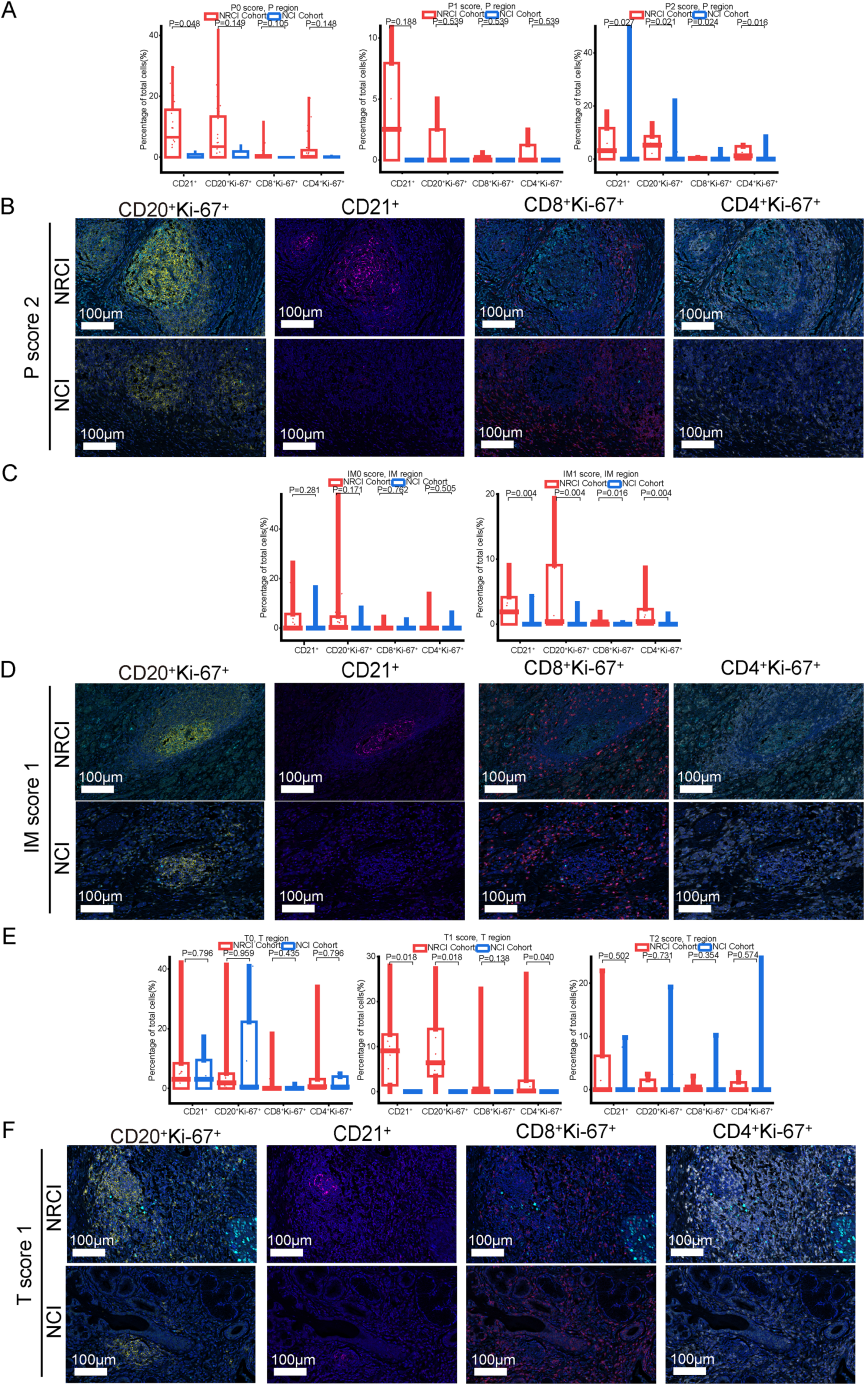
Differences in immune cell composition of M-TLSs in the NRCI and NCI cohorts under identical TLS scores (P score, IM score, and T score). **(A)** Quantification of M-TLSs in the NRCI and NCI cohorts with identical P scores, focusing on the percentages of CD20^+^Ki-67^+^ B cells, CD21^+^ DCs, CD8^+^Ki-67^+^ cytotoxic T lymphocytes, and CD4^+^Ki-67^+^ Th cells in peritumoral regions. **(B)** Representative mIHC images showing staining of CD20, CD21, Ki-67, CD8, and CD4 in M-TLSs with a P score of 2 in the NRCI and NCI cohorts. **(C)** Quantification of M-TLSs in the NRCI and NCI cohorts with identical IM scores, focusing on the percentages of CD20^+^Ki-67^+^ B cells, CD21^+^ DCs, CD8^+^Ki-67^+^ cytotoxic T lymphocytes, and CD4^+^Ki-67^+^ Th cells in invasive margins. **(D)** Representative mIHC images showing staining of CD20, CD21, Ki-67, CD8, and CD4 in M-TLSs with an IM score of 1 in the NRCI and NCI cohorts. Scale bar, 100 µm. **(E)** Quantification of M-TLSs in the NRCI and NCI cohorts with identical T scores, focusing on the percentages of CD20^+^Ki-67^+^ B cells, CD21^+^ DCs, CD8^+^Ki-67^+^ cytotoxic T lymphocytes, and CD4^+^Ki-67^+^ Th cells in intratumoral regions. **(F)** Representative mIHC images showing staining of CD20, CD21, Ki-67, CD8, and CD4 in M-TLSs with a T score of 1 in the NRCI and NCI cohorts. Scale bar, 100 µm. mIHC, multiplex immunohistochemistry; M-TLSs, mature tertiary lymphoid structures; DCs, dendritic cells.

Next, an analysis was performed to evaluate the impact of different treatments on the immune cell composition of M-TLSs under identical TLS scores. When the P score was 2, more CD20^+^Ki-67^+^ B cells, CD21^+^ cells, CD8^+^Ki-67^+^ cytotoxic T lymphocytes, and CD4^+^Ki-67^+^ Th cells were observed in the NRCI group compared to the NCI group in the peritumoral region (**Figures 6A, B**, percentage: all P<0.05). At a P score of 0, the proportion of CD21^+^ cells was higher in the NRCI group than in the NCI group, while the other three types of immune cells showed no significant differences between the two groups (**Figure 6A**; **Supplementary Figure 4E**). At a P score of 1, there were no significant differences in the immune cell composition of M-TLSs between the NRCI and NCI groups (**Figure 6A**, percentage: all *P*>0.05). In the invasive margin, the proportions of CD20^+^Ki-67^+^ B cells, CD21^+^ cells, CD8^+^Ki-67^+^ cytotoxic T lymphocytes, and CD4^+^Ki-67+ Th cells were higher in the NRCI group than in the NCI group when the IM score was 1 (**Figures 6C, D**, percentage: all *P*<0.05). At an IM score of 0, there were no significant differences between the NRCI and NCI groups (**Figure 6C**, percentage: all *P*>0.05). However, in the tumor region, the proportions of CD20^+^Ki-67^+^ B cells, CD21^+^ cells, and CD4^+^Ki-67^+^ Th cells were higher in the NRCI group than in the NCI group when the T score was 1 (**Figures 6E, F**, percentage: all *P*<0.05). When the T score was 0 or 2, there were no significant differences in the immune cell composition of M-TLSs between the NRCI and NCI groups (**Figure 6E**, percentage: all *P*>0.05). This suggested that at higher TLSs scores, NRCI more effectively activated immune responses by the activation and infiltration of immune cells in M-TLSs. Interestingly, at T scores of 0 to 2, the proportion of CD8^+^Ki-67^+^ cytotoxic T lymphocytes did not differ significantly between the NRCI and NCI groups (**Figure 6E**, percentage: all *P*>0.05). It suggested that when M-TLSs abundance was comparable, the predominant cells mediating antitumor immunity in M-TLSs within the tumor region were proliferating CD20^+^ B cells, CD21^+^ cells, and CD4^+^ Th cells, rather than proliferating CD8^+^ cytotoxic T lymphocytes.

To explore the potential mechanisms underlying the opposing prognostic trends of intra-tumoral and peri-tumoral TLSs, we analyzed 40 ESCC cases from the NRCI cohort and evaluated differences in their immune-cell composition. No statistically significant differences were observed in the percentages of CD20^+^Ki-67^+^ B cells, CD21^+^ follicular dendritic cells, CD8^+^Ki-67^+^ cytotoxic T cells (within total CD8^+^), or CD4^+^Ki-67^+^ helper T cells (within total CD4^+^) between intra-tumoral and peri-tumoral TLSs (**Supplementary Figure 5A**; all *P*>0.05). Next, we assessed the association between TLS scores and immune-cell composition. The results indicated that, within intra-tumoral TLSs, the proportions of CD20^+^Ki-67^+^ B cells, CD21^+^ cells, CD8^+^Ki-67^+^ cytotoxic T cells, and CD4^+^Ki-67^+^ helper T cells did not differ significantly across T score subgroups (0 to 2). Similarly, in peri-tumoral TLSs, these immune-cell subsets showed no significant differences across P score subgroups (0 to 2) (**Supplementary Figures 5B, C**; all *P*>0.05). These findings suggested that the aforementioned immune cells might exert comparable anti-tumor effects within both intra-tumoral and peri-tumoral TLSs. The association between high peri-tumoral TLS abundance and poorer prognosis may not be attributable to these specific cell types, but rather may reflect the influence of other cellular components in the peri-tumoral region that contribute to an immunosuppressive microenvironment, the mechanisms of which warrant further investigation.

## 4 Discussion

It remains unclear what the role of TLSs is in ESCCs treated with NRCI. Previous work by Huang et al. demonstrated that the presence of M-TLSs within resected tumors was associated with prolonged survival in ESCCs across various neoadjuvant treatment regimens (16). However, the study did not include the NRCI regimen. We analyzed the abundance and maturity of TLSs in resectable ESCCs treated with NRCI or NCI, and compared the differences in M-TLSs between the two treatment groups. To our knowledge, this is the first study to reveal the correlation between the abundance and maturity of TLSs and OS in resectable ESCCs treated with NRCI.

**Figure 7:**
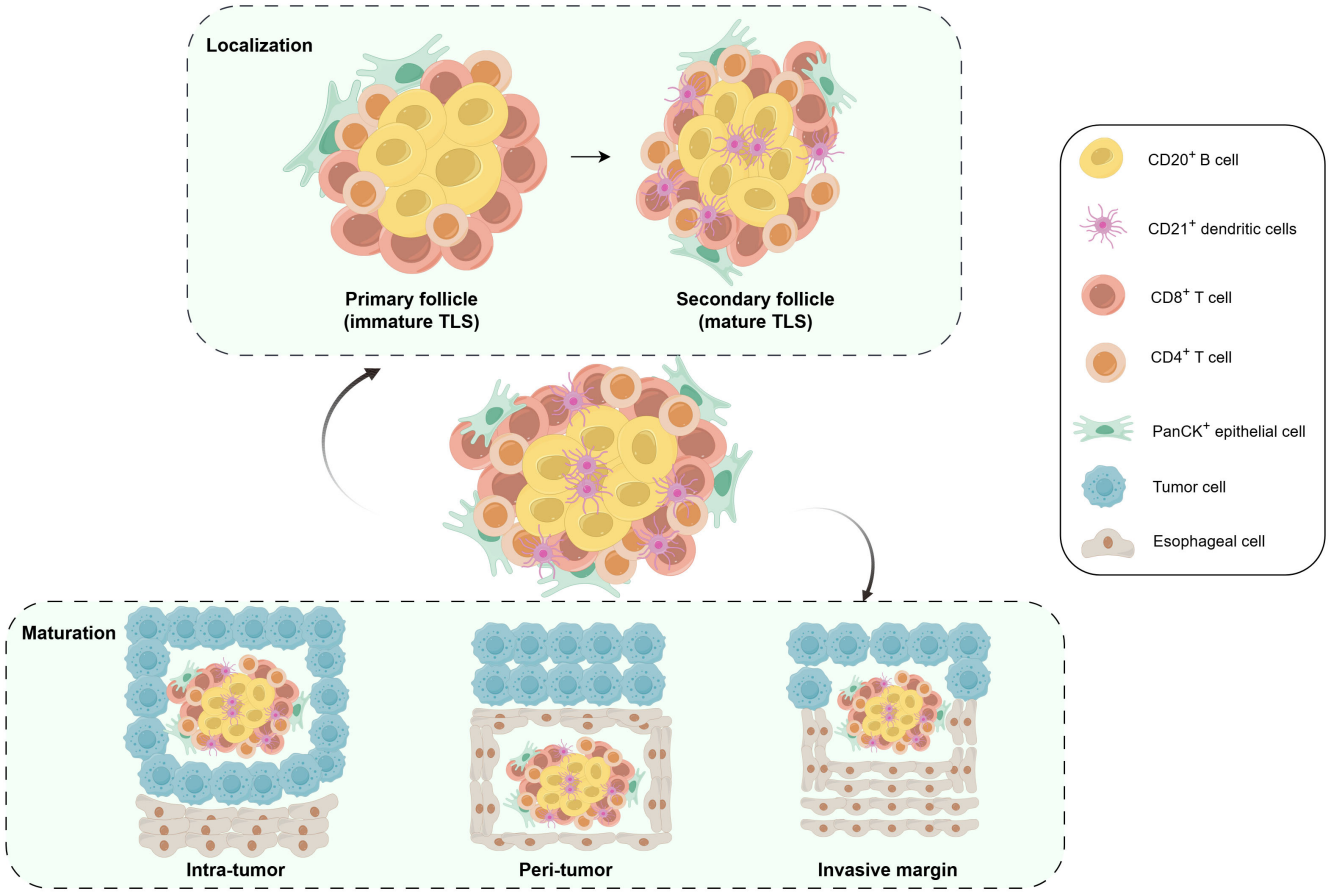
Schematic illustrating the distribution and cellular composition of TLSs in ESCC. Schematic illustrating the distribution and cellular composition of TLSs in ESCC. mIHC, multiplex immunohistochemistry; M-TLSs, mature tertiary lymphoid structures; DCs, dendritic cells.

It remains largely unknown whether NRCI affects TLSs formation in resectable ESCCs. Our study found that the NRCI group had longer OS and DFS, along with a higher MPR rate, compared to the NCI group. Meanwhile, OS and DFS were significantly longer in the MPR group compared to the Non-MPR group. This finding aligned with recent studies, particularly regarding the role of radiotherapy in enhancing immunotherapy efficacy. The improved MPR rate may result from a synergistic effect between the direct cytotoxicity of radiotherapy against tumor cells and its immune-activating function. A Study has shown that radiotherapy can directly induce DNA double-strand breaks in tumor cells, leading to cell death and loss of proliferative capacity (24). Meanwhile, radiotherapy also modulates the tumor microenvironment and promotes immune activation. Such as, Zhang et al., Yan et al., Li et al., and Jiang et al. have all reported that NRCI therapy enhances immune responses within the tumor microenvironment, thereby improving the prognosis of ESCC (25–28). The synergistic effect effectively reduced tumor burden and significantly contributed to the increased MPR rate observed in our NRCI-treated cohort. Our study further demonstrated that NRCI therapy offered better clinical outcomes for ESCCs compared to NCI therapy. A strong association has been identified between the presence of TLSs and favorable survival in various cancer types (22, 29, 30). However, the correlation between the abundance and maturity of TLSs and OS in resectable ESCCs treated with NRCI has not been explored. We demonstrated the distinct roles of TLSs based on their distribution, abundance, and maturity in tumors and surrounding regions (**Figure 7**). In the NRCI group, the abundance of TLSs within tumors and invasive margins was a marker of the favorable prognosis, while the abundance of peritumoral TLSs was associated with a poor prognosis, consistent with findings in the NCI group. This suggested that the abundance of TLSs exerted varying immune functions across different tissue regions. Notably, the abundance of peritumoral TLSs was identified as an independent predictor of OS in both cohorts.

The maturity of TLSs was an essential parameter of tumor immune structures, with a potential predictive value in ESCC (29, 31–33). Our study showed that patients with a higher maturity of TLSs within tumors had better OS in both cohorts. In the NRCI group, the high maturity of TLSs at invasive margins also indicated the better OS, whereas high maturity of peritumoral TLSs was associated with the poorer OS. These findings further supported that TLSs may have region-specific immune functions in different tissue areas, especially with NRCI therapy. In the NCI cohort, the TLSs maturation stage in the T region showed results consistent with those from the NRCI cohort. Interestingly, ESCCs with E-TLSs had a shorter OS compared to TLS-negative and M-TLSs ESCCs in the P region, whereas ESCCs with E-TLSs had a prolonged OS in the IM region. We speculated that those findings stemmed from NRCI enhancing M-TLS activation in the tumor and its microenvironment, thereby improving the combined efficacy of radiotherapy and immunotherapy and boosting the patient’s anti-tumor immune response. In contrast, NCI may weaken the anti-tumor immune effect of M-TLSs in the invasive margin and adjacent normal tissues. Moreover, this was consistent with the findings from the NRCI group showing superior results in key prognostic indicators, including OS, DFS, and MPR rate, compared to the NCI group. In addition, it was observed that there were no significant associations between subregions and TLS maturity stages in the NRCI cohort, in contrast to the NCI cohort. These findings indicated that NRCI enhanced the independence of TLS maturity stages across subregions. We hypothesize that this phenomenon arises because NRCI achieves more precise targeting of tumor regions compared to NCI, thereby promoting more independent maturity characteristics of TLSs in the tumor regions, invasive margins, and peritumoral areas. Huang et al. and Helmink et al. found that in responders to neoadjuvant immunotherapy, the number of TLSs dominated by M-TLSs increased in both ESCC and melanoma (16, 34). Currently, there are no reports on animal models exploring the effects of neoadjuvant immunotherapy on the formation and maturation of TLSs in resectable ESCC. However, it has been shown that immunotherapy induced the formation and maturation of TLSs in mouse models of non-small cell lung cancer and melanoma (35, 36). Additionally, Helmink et al. reported that the mice with lung cancers treated with anti-PD-1 therapy showed significantly increased TLSs abundance and organization compared to control groups (35). Therefore, the impact of NRCI on TLSs formation and maturation seems not a simple additive or synergistic effect, as immunotherapies such as anti-PD-1 may induce TLSs maturation in ESCC, while chemotherapy and radiotherapy could impair TLSs abundance and maturity. Our study suggested that inducing TLSs appears to be a potential anticancer mechanism of NRCI therapy for resectable ESCC, warranting further investigation into its biological mechanisms.

It had been shown that radiotherapy-induced immunogenic cell death (ICD) enhanced the immune system’s recognition and clearance of tumors by releasing tumor-associated antigens or altering the presentation of tumor antigens (37–40). Meanwhile, radiotherapy modulated the tumor microenvironment to promote immune cell mobilization, activation, and infiltration, thereby enhancing the efficacy of immunotherapy (27, 41). Consistent with previous studies, our findings demonstrated that the NRCI group exhibited higher proportions of CD20^+^Ki-67^+^ B cells, CD21+ DCs, CD8^+^Ki-67^+^ cytotoxic T cells, and CD4^+^Ki-67^+^ Th cells within M-TLSs compared to the NCI group under identical sub-regional conditions (intratumoral, invasive margin, and peritumoral areas). This indicated that NRCI therapy leveraged radiotherapy to markedly boost immune cell activation, proliferation, and infiltration within M-TLSs, thereby enhancing anti-tumor immune responses. It has been found that NRCI indirectly inhibited tumor cell proliferation by enhancing immune responses in the ESCC tumor microenvironment, thereby diminishing tumor aggressiveness and metastasis (42). Our investigation observed that the proportion of proliferating PanCK^+^Ki-67^+^ epithelial cells in M-TLSs was lower in the NRCI group than in the NCI group. This finding further validated the pivotal role of radiotherapy in augmenting immune responses within NRCI therapy, illustrating its superiority over NCI.

A recent study found that NCI enhanced the abundance and maturity of TLSs through immunotherapy compared to initial treatment and neoadjuvant chemotherapy, but did not find significant differences in M-TLSs among different treatment groups (43). In contrast, our findings revealed that the NRCI group showed elevated proportions of CD20^+^Ki-67^+^ B cells, CD21^+^ DCs, CD8^+^Ki-67^+^ cytotoxic T cells, and CD4^+^Ki-67^+^ Th cells in regions with high TLS scores (P score=2, IM score=1). This suggested that NRCI therapy induced stronger immune responses in areas with high TLS scores, whereas NCI alone failed to enhance immune cell activation and proliferation. For resectable ESCC, it remains unclear how the elevated P scores result in decreased survival rates. Chen et al. found that elevated P scores were accompanied by increased CD4^+^ Foxp3^+^ Treg cells and CD68^+^ CD163^+^ M2 macrophages, correlating with poor survival in colorectal liver metastases (44). We observed that ESCCs with a P score of 2 exhibited a poor prognosis regardless of treatment, and NRCI therapy increased the proportions of CD20^+^Ki-67^+^ B cells, CD21^+^ DCs, CD8^+^Ki-67^+^ cytotoxic T cells, and CD4^+^Ki-67^+^ Th cells in M-TLSs compared to NCI therapy. This suggested that increasing these immune cell proportions through NRCI therapy to enhance M-TLS activation and anti-tumor effects while counteracting the immunosuppressive effects of CD4^+^ Foxp3^+^ Treg cells and CD68^+^ CD163^+^ M2 macrophages might be a highly promising neoadjuvant strategy to extend patient survival. Additionally, when the T score of the tumor region was 2, there was no significant difference in immune cell activation and infiltration within M-TLSs between the two treatment groups, which may be related to the saturation of immune cell infiltration.

It is clear that the activation of B cells and DCs is a critical factor in anti-tumor immune responses in M-TLSs (45–48). When M-TLSs abundance was the same, there was no significant difference in CD8^+^Ki-67^+^ cytotoxic T cells within tumor-region M-TLSs between the two treatment groups, whereas the proportions of proliferating and activated CD20^+^Ki-67^+^ B cells, CD21^+^ DCs, and CD4^+^Ki-67^+^ Th cells were higher in the NRCI group. This indicated that the primary effector cells of the anti-tumor immune response were proliferative and activated CD20^+^ B cells, CD21^+^ DCs, and CD4^+^ Th cells, rather than CD8^+^ cytotoxic T cells within tumor-region M-TLSs.

To explore the mechanisms underlying the opposing prognostic significance of intra-tumoral versus peri-tumoral TLSs, we analyzed the immune-cell composition of intra- and peri-tumoral TLSs in the NRCI cohort. Overall, we found no significant differences in the distribution of CD20^+^Ki-67^+^ B cells, CD21^+^ follicular dendritic cells, CD8^+^Ki-67^+^ cytotoxic T cells, or CD4^+^Ki-67^+^ Th cells between intra-tumoral and peri-tumoral TLSs. The composition of these immune cells also did not change significantly with either T score in the tumor or P score in the peritumoral region. This suggested that the aforementioned immune cell populations may exert similar anti-tumor effects in both intra- and peri-tumoral TLSs. Therefore, the association between high peri-tumoral TLS abundance and poor prognosis may be more likely attributable to the presence of other immunosuppressive cell subsets, such as, recent studies have shown that peri-tumoral TLSs may be infiltrated by immunosuppressive cells such as M2 macrophages and regulatory T cells, resulting in their functional inhibition (44, 49). Such mechanisms may partly account for the observed correlation between peri-tumoral TLSs and unfavorable clinical outcomes.

However, several phenomena and mechanisms require further investigation. Firstly, the sample size is relatively small, particularly in the NRCI group (n=49); thus, larger cohort studies should be conducted in the future to validate our conclusions. Secondly, as all retrospective studies have inherent limitations, prospective clinical trials should be conducted in the future to validate the potential value of TLSs abundance and maturity as biomarkers predicting OS and immune therapy response in ESCC. Thirdly, the underlying mechanisms by which NRCI therapy enhances the expansion and activation of M-TLSs to improve patient prognosis remain to be further elucidated. Further clarification is needed regarding the mechanisms underlying the differences in immune cell activation and infiltration within M-TLSs between the NRCI and NCI groups. Fourth, as this study is a single-center cohort, future research should include multi-center cohort studies for external validation to further confirm our conclusions. Moreover, a recent study has indicated that TLSs in hepatocellular carcinoma exhibit significant heterogeneity in gene expression patterns and spatial distribution of immune cells (50). Therefore, future research should employ high-resolution spatiotemporal omics technologies to further investigate the spatial heterogeneity of TLSs in ESCC patients undergoing NRCI therapy. Sixth, further investigation is needed to define the precise immunosuppressive pathways and cellular constituents in the peritumoral microenvironment that contribute to the link between peri-tumoral TLSs and unfavorable prognosis, thereby clarifying the exact role of TLSs in the tumor immune landscape. Finally, future studies should further explore whether the cellular composition of TLSs undergoes dynamic changes during tumor progression and NRCI treatment, and whether external interventions, such as adjusting radiation dosage or neoadjuvant therapy cycles, can modulate the cellular composition of TLSs to enhance anti-tumor immune responses.

## 5 Conclusion

This study demonstrated that NRCI therapy prolonged patient survival and markedly increased the MPR rate compared to NCI therapy. Radiotherapy enhanced treatment efficacy by strengthening the immune response to immunotherapy. Moreover, the distribution, abundance, and maturity of TLSs were closely associated with OS in ESCCs, with the abundance of peritumoral TLSs identified as an independent predictor of OS. Meanwhile, the abundance of TLSs exerted different immune effects in distinct tissue regions: the abundance of intratumoral TLSs and the abundance of TLSs in invasive margins were indicators of the favorable prognosis, whereas the abundance of peritumoral TLSs was associated with a poor prognosis. Notably, this study revealed that the specific cellular composition of M-TLSs had a significant impact on the prognosis of ESCCs, particularly the proliferative and activated CD20^+^Ki-67^+^ B cells, CD21^+^ DCs, and proliferative and activated CD4^+^Ki-67^+^ Th cells. Those cells could be utilized to enhance the clinical response to NRCI therapy, highlighting their potential clinical applications.

## Data Availability

Original datasets are available in a publicly accessible repository: the data presented in the study are deposited in the China National Center for Bioinformation - National Genomics Data Center repository (https://ngdc.cncb.ac.cn), accession number PRJCA044496.
