# Supplementary material for: Tertiary lymphoid structures correlate with the therapeutic efficacy and prognosis of resectable esophageal squamous cell carcinoma undergoing neoadjuvant chemoradiotherapy plus immunotherapy
Source: Front Immunol. 2025 Aug 22;16:1616247. doi: 10.3389/fimmu.2025.1616247 (PMC12411778; doi:10.3389/fimmu.2025.1616247)
Supplement: Supplementary file 1 [file DataSheet1.docx]

Supplementary Material

Table of contents

Supplementary Methods…………………………………………………2

Supplementary Figures…………………………………………………..7

Supplementary Tables……………………………………………………12

# 1 Supplementary Methods

**1.1 Inclusion Criteria**

Inclusion Criteria as shown in Supplementary Figure 1.

**1.2 Quantification of TLSs abundance in the IM region (IM score)**The subregions of the whole slide images are illustrated in Supplementary Figure 2A. The density of TLSss in the IM region (IM score) was divided into two groups: (1) Score 0: No TLSss in the IM region. (2) Score 1: At least one TLSs in the IM region, either convergent or non-convergent across the entire IM area (Supplementary Figure 2B).

**1.3 Verification of the TLSs scoring system**To ensure the accuracy of the TLSs scoring system, pathologists evaluated the TLSs distribution in the radiochemotherapy plus immunotherapy (NRCI) cohort, and TLSs scores for the T and IM regions were calculated based on the number of TLSs divided by the area of the scoring subregion. As shown in Supplementary Figure 2C, TLSs scores were mapped to a relative scoring system of 0–100%. The maximum and minimum values of TLSs density were defined as Densitymax and Densitymin, serving as the upper and lower limits of the 0–100% mapping. The density value of each scoring subregion (Densityi) was mapped to a relative score using the following formula: Relative Scorei=（Densityi−Densitymin）/（Densitymax−Densitymin）×100%. The subregion with the highest density received a relative score of 100%, while the subregion with the lowest density received a relative score of 0%. For T scores, values of 0, 1, and 2 corresponded to TLSs densities of 0–9.7%, 9.7–100%, respectively. For IM scores, a value of 0 corresponded to a TLSs density of 0%, while a score of 1 corresponded to TLSs densities ranging from 0% to 100%. These findings further validate that the TLSs scoring system represents TLSs density accurately.

# 2 Supplementary Figures

**
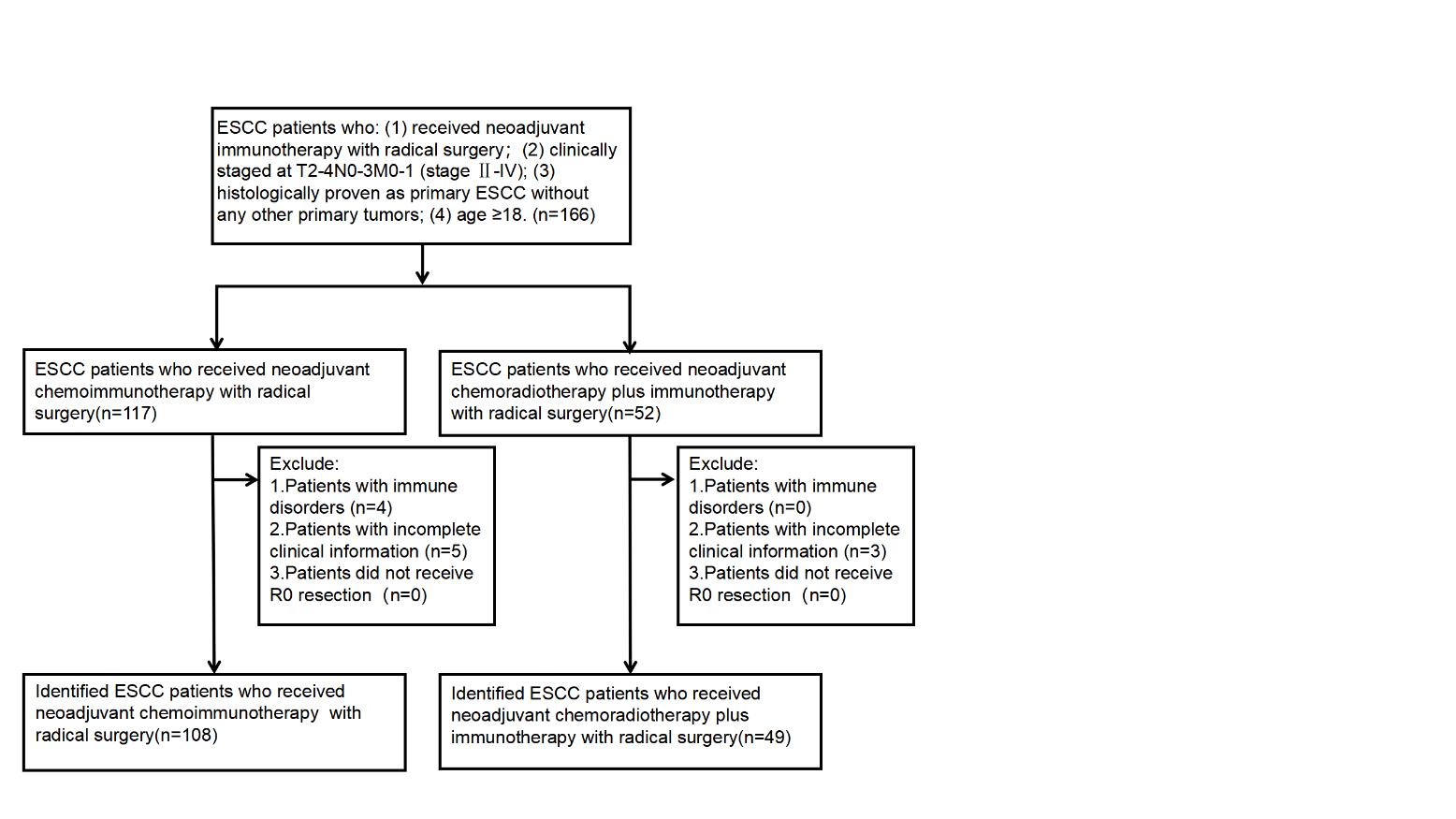
**

**Supplementary Figure 1. Flow chart for the inclusion and exclusion of ESCC patients and analysis of tumor information. ESCC, esophageal squamous cell cancer.**


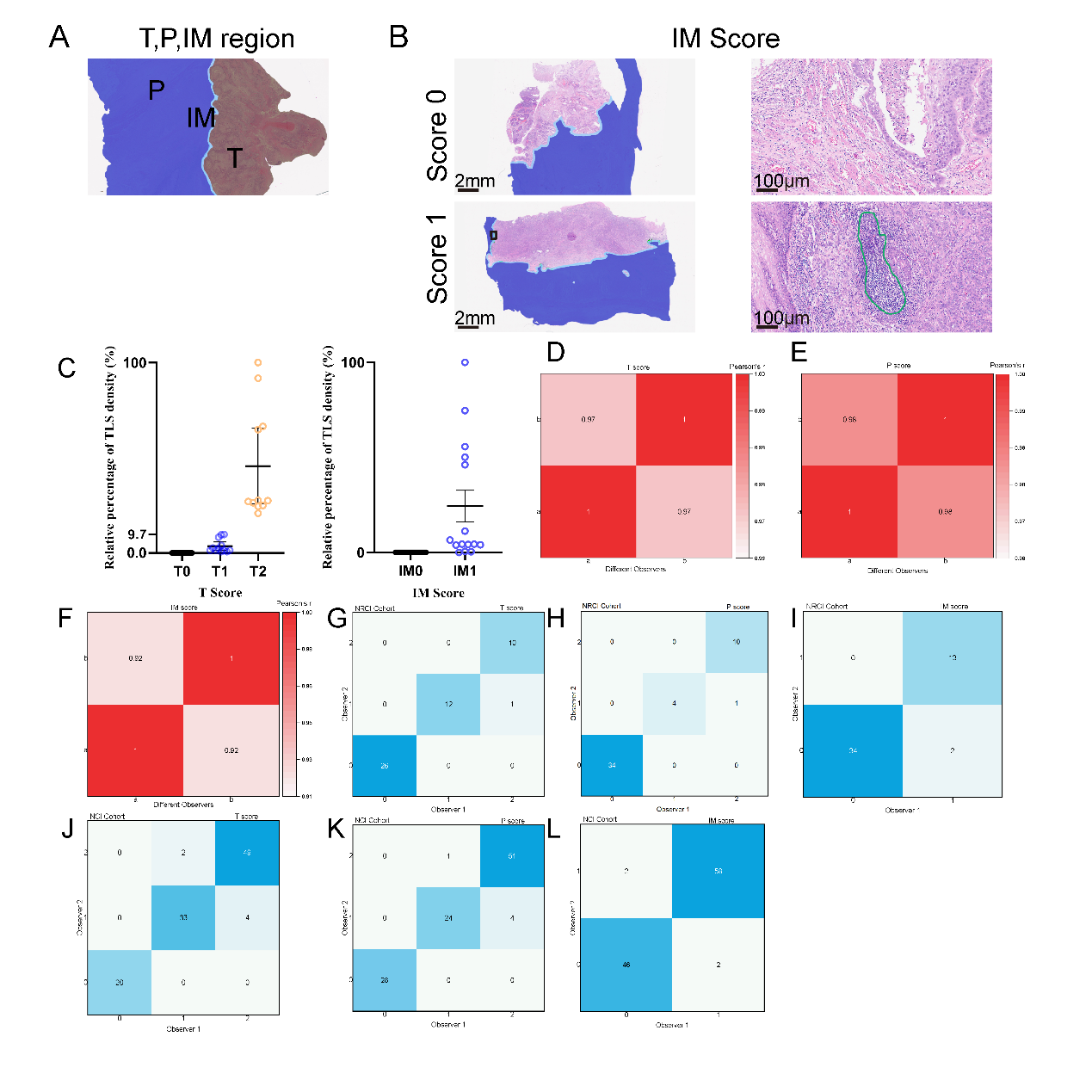


**Supplementary Figure 2. Additional information on the TLS scoring system.** (A, B) Representative whole slide images illustrating intratumoral (T), peritumoral (P), and invasive margin (IM) regions. Intratumoral areas are highlighted in light orange, peritumoral areas in dark blue, and invasive margin areas in light blue. (C) Distribution of TLS scores across different T and IM scores in the NRCI cohort. TLS scores were calculated as the number of TLSs divided by the area of the scoring subregion. (D-F) Correlation matrices demonstrating the reproducibility of the TLS scoring system between two independent observers (denoted as a and b) in the NCI cohort. (G-L) Detailed T, P, and IM scores in the NRCI cohort (G-I); detailed T, P, and IM scores in the NCI cohort (J-L).


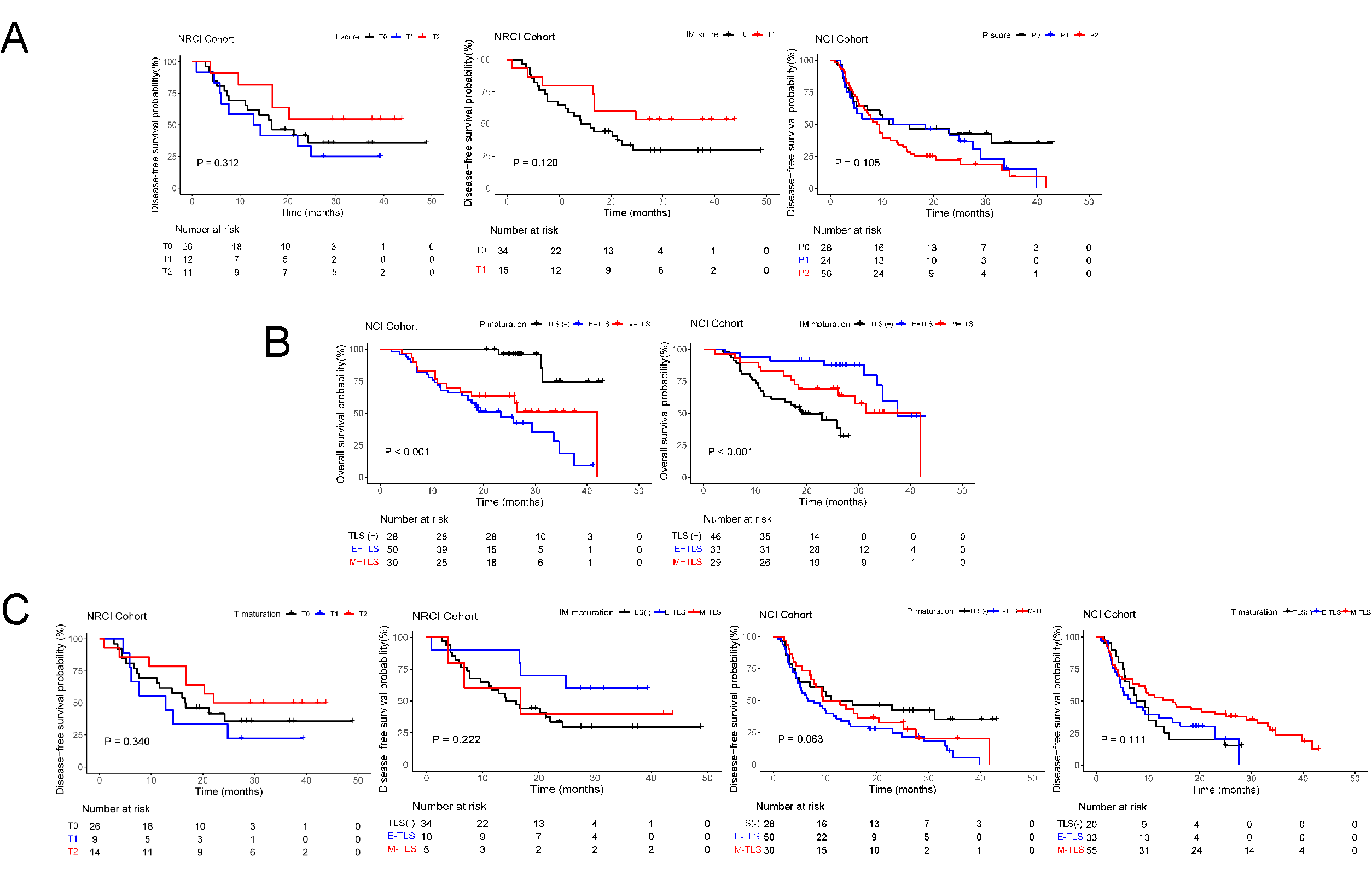


**Supplementary Figure 3. Additional Kaplan-Meier analysis of the association between TLS score, TLS maturation with OS and DFS in the NRCI and NCI cohorts.** (A) Kaplan-Meier curves illustrating DFS in the NRCI cohort stratified by T and IM scores, and DFS in the NCI cohort stratified by P score. (B) Kaplan-Meier curves illustrating OS in the NCI cohort stratified by P and IM maturation. (C) Kaplan-Meier curves illustrating DFS in the NRCI cohort stratified by T and IM maturation, OS in the NCI cohort stratified by P maturation, and DFS in the NCI cohort stratified by T maturation.


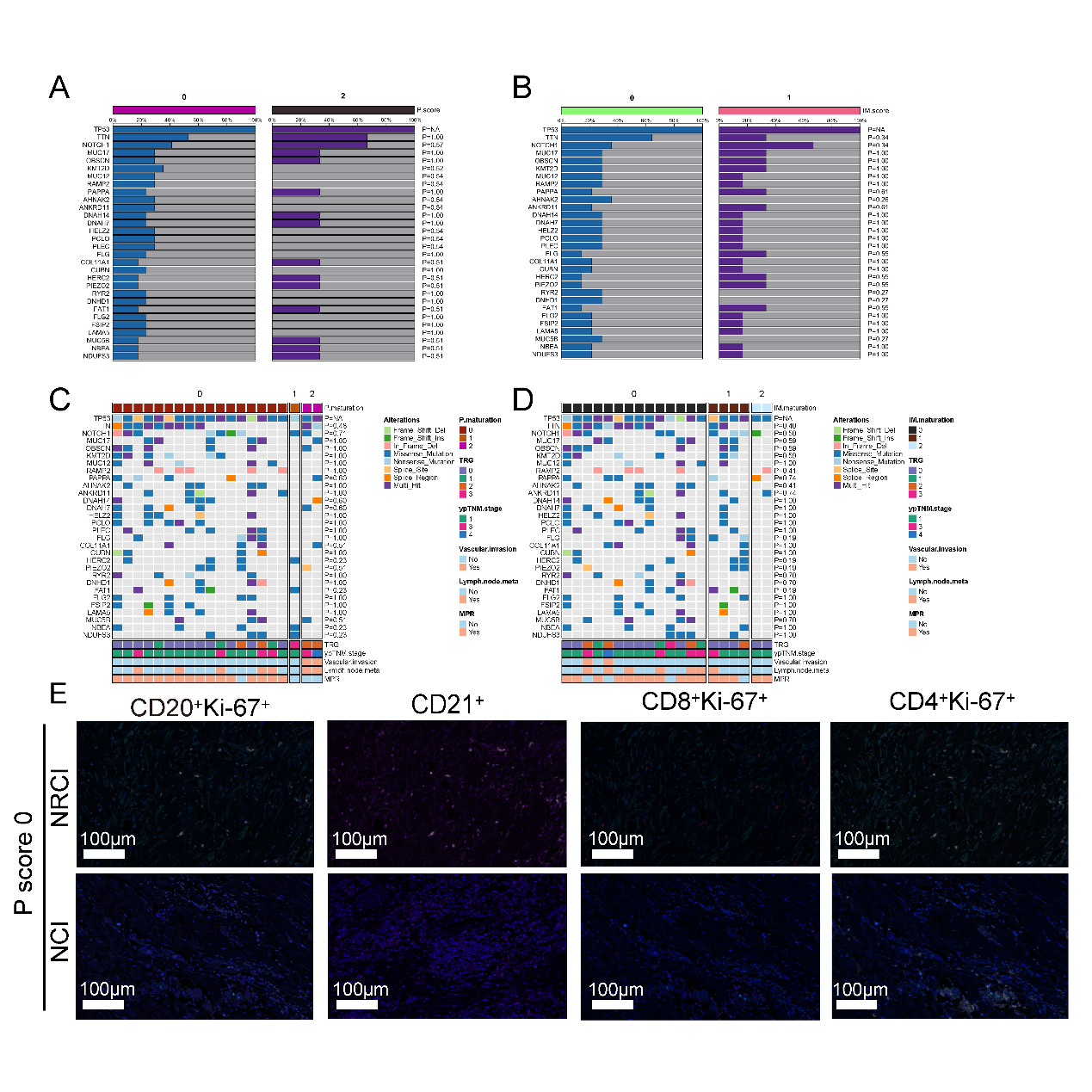


**Supplementary Figure 4. Correlation analysis of TLS infiltration, maturation, and high-frequency mutations in ESCCs.** (A-D) No significant correlations were observed between high-frequency mutations and P score (A), IM score (B), P maturation (C), or IM maturation (D) in ESCCs (all *P*> 0.05). (E) Representative mIHC images showing staining for CD20, CD21, Ki-67, CD8, and CD4 in M-TLSs from the NRCI and NCI cohorts when the P score was 0. Scale bar, 100 μm. mIHC, multiplex immunohistochemistry; M-TLSs, mature tertiary lymphoid structures.
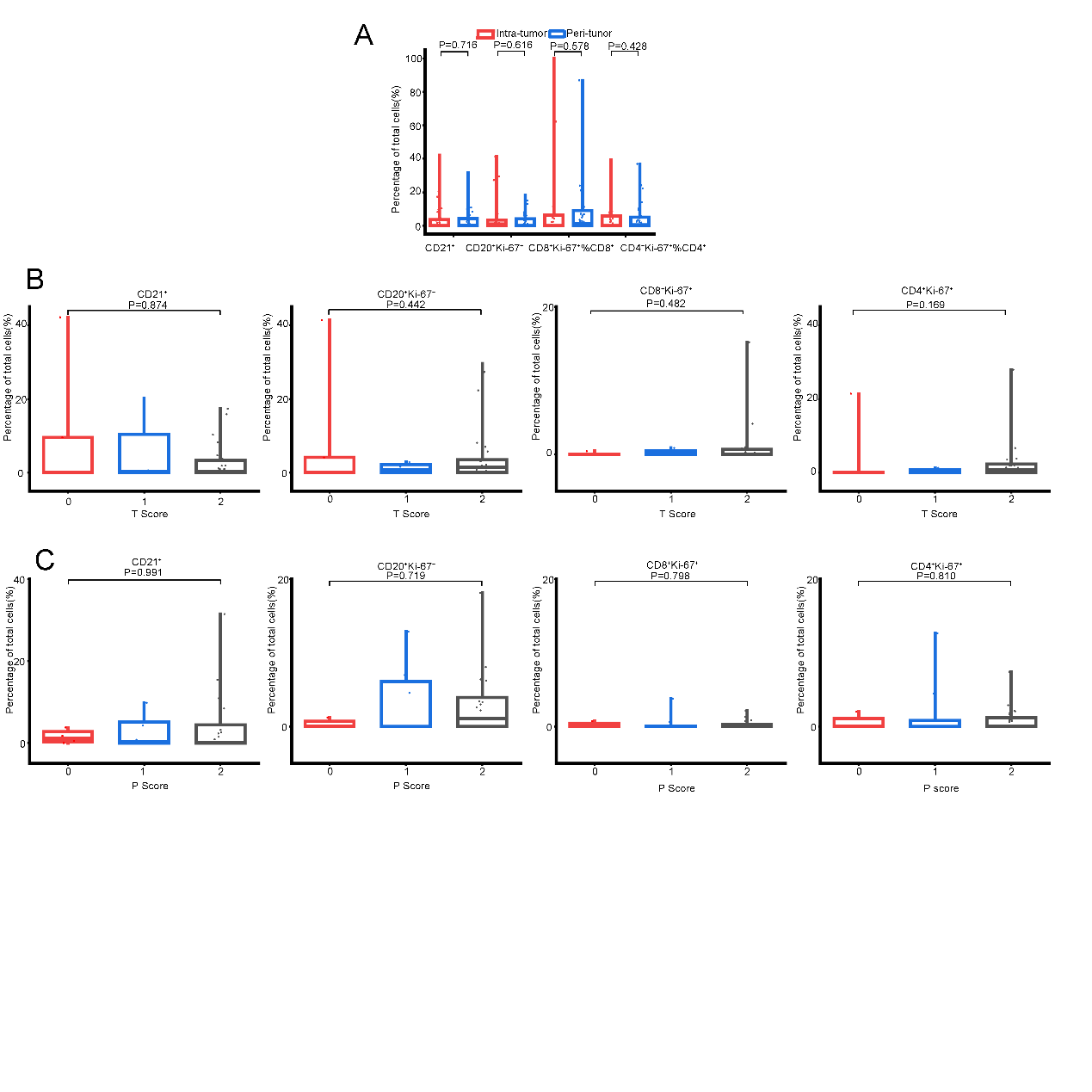


**Supplementary Figure 5. Immune‐cell composition of intratumoral and peritumoral TLSs in the NRCI cohort.** (A) Percentages of CD21⁺ follicular dendritic cells, proliferating B cells (CD20⁺Ki-67⁺), proliferating cytotoxic T cells (CD8⁺Ki-67⁺, expressed as % of total CD8⁺), and proliferating helper T cells (CD4⁺Ki-67⁺, expressed as % of total CD4⁺) within mature TLSs located intra-tumorally versus peri-tumorally. No significant differences were observed for any subset (all *P*> 0.05). (B) Immune-cell composition of intra-tumoral TLSs stratified by tumour-region TLS abundance (T score 0, 1, 2). No significant differences among T score subsets (all *P*> 0.05). (C) Immune-cell composition of peri-tumoral TLSs stratified by peri-tumoral TLS abundance (P score 0, 1, 2). No significant differences among P score subsets (all *P*> 0.05).

## 3 Supplementary Tables

| **Supplementary Table 1. Information on the antibodies used in our research** | | |
| --- | --- | --- |
| Antibodies | Source | Catalogue or clone |
| Ki-67 | CST | #9449 |
| PanCK | Abcam | ab308262 |
| CD20 | CST | #48750 |
| CD21 | CST | #76069 |
| CD8 | Zsbio | ZA-0508 |
| CD4 | Abcam | ab133616 |

| **Supplementary Table 2.Univariate and multivariate analysis of DFS for the two different groups** | | | | | | | | |
| --- | --- | --- | --- | --- | --- | --- | --- | --- |
| **Variables** | NCI cohort（n=108) | |  |  | NRCI cohort(n=49) | |  |  |
|  | Univariate  analysis |  | Multivariate analysis |  | Univariate  analysis |  | Multivariate analysis |  |
|  | *HR* (95%*CI*) | *P* value | *HR* (95%*CI*) | *P* value | *HR* (95%*CI*) | *P* value | *HR* (95%CI) | *P* value |
| **Gender**  **(Male or female)** | 0.81(0.37-1.76) | 0.664 |  |  | 0.51（0.18-1.46） | 0.349 |  |  |
| **Age,years**  **＜64 vs ≥64** | 0.95(0.62-1.47) | 0.867 |  |  | 0.67（0.27-1.64） | 0.437 |  |  |
| **MPR**  **（No vs. Yes)** | 0.50(0.32-0.79) | 0.040 | 0.49(0.23-1.04) | 0.679 | 0.51（0.23-1.11） | 0.216 |  |  |
| **AJCC/UICC 8th cTNM stage** |  | 0.599 |  |  |  | 0.411 |  |  |
| **Ⅱ** | Reference |  |  |  | Reference |  |  |  |
| **Ⅲ** | 0.97(0.59-1.59) | 0.930 |  |  | 2.08（0.78-5.56） | 0.281 |  |  |
| **Ⅵ** | 1.40(0.73-2.69) | 0.450 |  |  | 2.17（0.62-7.57） | 0.349 |  |  |
| **AJCC/UICC 8th ypTNM stage** |  | 0.108 |  | 0.927 |  | 0.010 |  | 0.425 |
| **Ⅰ** | Reference |  |  |  | Reference |  | Reference |  |
| **Ⅱ** | 1.65(0.87-3.13) | 0.221 | 0.96(0.38-2.42) | 0.927 | 1.05（0.14-8.10） | 0.961 | 2.40(0.15-38.55) | 0.699 |
| **Ⅲ** | 1.51(0.92-2.51) | 0.211 | 0.90(0.43-1.87) | 0.927 | 6.50（2.76-15.29） | 0.010 | 6.21(0.88-43.61) | 0.286 |
| **Ⅵ** | 3.09(1.42-6.74) | 0.040 | 1.46(0.53-4.00) | 0.927 | 7.18（2.35-21.89） | 0.010 | 7.68(1.10-53.79) | 0.260 |
| **Mild-to-moderate  myelosuppression**  **（No vs. Yes)** | 0.49(0.21-1.13) | 0.206 |  |  | 1.43（0.69-2.99） | 0.412 |  |  |
| **Neoadjuvant therapy no of cycles**  **(1 vs. ≥2)** | 0.58(0.14-2.39) | 0.599 |  |  | 1.06（0.48-2.31） | 0.918 |  |  |
| **Lymph node metastasis**  **（No vs. Yes)** | 1.49(0.97-2.31) | 0.185 |  |  | 3.87（1.83-8.22） | 0.010 | 3.58(1.16-11.06) | 0.260 |
| **Differentiation** |  | 0.797 |  |  |  | 0.288 |  |  |
| **Well** | Reference |  |  |  | - |  |  |  |
| **Moderate** | 1.26(0.54-2.93) | 0.664 |  |  | Reference |  |  |  |
| **Poor** | 1.42(0.57-3.52) | 0.599 |  |  | 1.97（0.86-4.53） | 0.288 |  |  |
| **Vascular invasion**  **（No vs. Yes)** | 1.36(0.79-2.32) | 0.412 |  |  | 4.96（1.74-14.16） | 0.020 | 1.33(0.23-7.63) | 0.811 |
| **Nerve infiltration**  **（No vs. Yes)** | 1.79(1.0-3.22) | 0.173 |  |  | 2.19（0.51-9.45） | 0.410 |  |  |
| **Tumor necrosis**  **(＜1.43 vs. ≥1.43)** | 1.83(0.73-4.58) | 0.323 |  |  | 1.68（0.79-3.54） | 0.327 |  |  |
| **TRG** |  | 0.599 |  |  |  | 0.170 |  | 0.700 |
| **0** | Reference |  |  |  | Reference |  | Reference |  |
| **1** | 1.27(0.64-2.54) | 0.599 |  |  | 2.29(0.88-5.96) | 0.216 | 0.66(0.18-2.40) | 0.699 |
| **2** | 1.65(0.92-2.95) | 0.206 |  |  | 3.84(1.46-10.12) | 0.034 | 0.27(0.03-2.26) | 0.425 |
| **3** | 1.77(0.95-3.30) | 0.185 |  |  | 3.44(0.89-13.31) | 0.206 | 0.86(0.11-6.98) | 0.885 |
| **TLSs maturation** |  |  |  |  |  |  |  |  |
| **T region** |  | 0.211 |  |  |  | 0.416 |  |  |
| **TLSs（-）** | Reference |  |  |  | Reference |  |  |  |
| **E-TLSs** | 0.99（0.53-1.83） | 0.961 |  |  | 1.38（0.56-3.35） | 0.538 |  |  |
| **M-TLSs** | 0.61（0.34-1.10） | 0.206 |  |  | 0.64(0.26-1.55) | 0.411 |  |  |
| **P region** |  | 0.185 |  | 0.927 |  | 0.043 |  |  |
| **TLSs（-）** | Reference |  |  |  | Reference |  |  |  |
| **E-TLSs** | 1.93（1.09-3.42） | 0.107 | 1.22(0.57-2.61) | 0.927 | 1.87（0.68-5.09） | 0.349 |  |  |
| **M-TLSs** | 1.41（0.75-2.65） | 0.425 | 1.04(0.48-2.25) | 0.927 | 3.73（1.58-8.85） | 0.020 |  |  |
| **IM region** |  | 0.040 |  | 0.679 |  | 0.363 |  |  |
| **TLSs（-）** | Reference |  |  |  | Reference |  |  |  |
| **E-TLSs** | 0.44（0.25-0.76） | 0.040 | 0.52(0.25-1.10) | 0.679 | 0.40（0.14-1.17） | 0.216 |  |  |
| **M-TLSs** | 0.51（0.30-0.88） | 0.086 | 0.54(0.23-1.28) | 0.679 | 0.82（0.25-2.75） | 0.794 |  |  |
| **TLSs abundence** |  |  |  |  |  |  |  |  |
| **T score** |  | 0.090 |  | 0.927 |  | 0.411 |  |  |
| **0** | Reference |  |  |  | Reference |  |  |  |
| **1** | 1.19（0.64-2.21） | 0.664 | 1.55(0.80-3.02) | 0.679 | 1.27（0.56-2.89） | 0.609 |  |  |
| **2** | 0.58（0.32-1.06） | 0.185 | 1.28(0.53-3.11) | 0.927 | 0.56（0.20-1.52） | 0.363 |  |  |
| **P score** |  | 0.211 |  | 0.927 |  | 0.078 |  | 0.425 |
| **0** | Reference |  |  |  | Reference |  | Reference |  |
| **1** | 1.48（0.77-2.86） | 0.387 | 0.93（0.51-1.69） | 0.927 | 2.02（0.66-6.03） | 0.349 | 0.25(0.03-1.79) | 0.425 |
| **2** | 1.83（1.04-3.21） | 0.135 | 1.33（0.54-3.16） | 0.927 | 3.16（1.38-7.21） | 0.033 | 1.82(0.52-6.45) | 0.572 |
| **IM score** |  | 0.040 |  | 0.679 |  | 0.192 |  |  |
| **0** | Reference |  |  |  | Reference |  |  |  |
| **1** | 0.51（0.32-0.81） | 0.040 | 1.07（0.48-2.38） | 0.679 | 0.45(0.19-1.05) | 0.192 |  |  |
| P-values between 0.05 and 0.10 were considered borderline significant.  P values were FDR-adjusted using the BH method.  Abbreviations: OS, overall survival; HR, hazard ratio; CI, confidence interval; NRCI, chemoradiotherapy plus immunotherapy; NCI, neoadjuvant chemoimmunotherapy; M-TLSs, highly mature TLSs; FDR, the false discovery rate; BH, Benjamini-Hochberg method. | | | | | | | | |
